# Soil Microbiome Dynamics During Pyritic Mine Tailing Phytostabilization: Understanding Microbial Bioindicators of Soil Acidification

**DOI:** 10.3389/fmicb.2019.01211

**Published:** 2019-06-05

**Authors:** John D. Hottenstein, Julie W. Neilson, Juliana Gil-Loaiza, Robert A. Root, Scott A. White, Jon Chorover, Raina M. Maier

**Affiliations:** Department of Soil, Water and Environmental Science, The University of Arizona, Tucson, AZ, United States

**Keywords:** phytostabilization, mine tailings, acid mine drainage, plant growth-promoting bacteria, iron-oxidizing bacteria, sulfur-oxidizing bacteria, iron-reducing bacteria, mine tailing acidification

## Abstract

Challenges to the reclamation of pyritic mine tailings arise from *in situ* acid generation that severely constrains the growth of natural revegetation. While acid mine drainage (AMD) microbial communities are well-studied under highly acidic conditions, fewer studies document the dynamics of microbial communities that generate acid from pyritic material under less acidic conditions that can allow establishment and support of plant growth. This research characterizes the taxonomic composition dynamics of microbial communities present during a 6-year compost-assisted phytostabilization field study in extremely acidic pyritic mine tailings. A complementary microcosm experiment was performed to identify successional community populations that enable the acidification process across a pH gradient. Taxonomic profiles of the microbial populations in both the field study and microcosms reveal shifts in microbial communities that play pivotal roles in facilitating acidification during the transition between moderately and highly acidic conditions. The potential co-occurrence of organoheterotrophic and lithoautotrophic energy metabolisms during acid generation suggests the importance of both groups in facilitating acidification. Taken together, this research suggests that key microbial populations associated with pH transitions could be used as bioindicators for either sustained future plant growth or for acid generation conditions that inhibit further plant growth.

## Introduction

Metal mining has left a lasting legacy of environmental degradation across the globe where a primary concern is the long-term generation of acid mine drainage (AMD) from residual mine wastes that contain large quantities of metal sulfide ores, such as pyrite (Schippers et al., 2000; Lu and Wang, 2012). Acidic mine tailings and AMD microbial communities have been well-characterized as being dominated by acidophilic microbial communities that catalyze iron and sulfur oxidation, thereby accelerating the dissolution of pyrite and the continual generation of acidity (Baker and Banfield, 2003; Schippers et al., 2010; Chen et al., 2016). However, the microbiology of moderately acidic mine tailings, such as those undergoing revegetation, has been less well-characterized. The kinetics of abiotic reactions involved in pyrite oxidation at circumneutral pH under aerobic conditions initially suggests that biotic activity plays a minimal role in acid generation before environmental conditions become acidic ((Bill) Evangelou and Zhang, 1995). However, microenvironments between soil aggregates create niches of varying oxygen availability and pH, where microbial activity may play a significant role in acid generation. Furthermore, experimental results suggest that biological activity can play an intricate role in increasing the rate of pyrite oxidation at circumneutral and moderately acidic pH conditions (Singer and Stumm, 1970; Moses and Herman, 1991; Ranjard and Richaume, 2001; Meruane and Vargas, 2003; Balci et al., 2007; Druschel et al., 2008; Luther et al., 2011).

Compost-assisted phytostabilization is the active establishment of vegetation in mine tailings after the addition of a compost soil amendment to create conditions conducive to plant growth, but challenges remain to prove its long-term efficacy (Mendez and Maier, 2008; Conesa et al., 2012). Sustained vegetative growth requires the establishment of a functioning soil substrate and the incorporation of soil amendments into pyritic mine wastes is an attempt to initiate the rapid development of these functional characteristics from undeveloped parent material (Zanuzzi et al., 2009; Li and Huang, 2015). Studies of mine tailings undergoing both natural and anthropogenic revegetation have attempted to capture the transition of both physical and biological properties from raw mine tailings to reclaimed soil substrates capable of supporting plant growth (Ye et al., 2000; Li et al., 2015, 2016a). Among lithotrophic populations in unamended mine tailings, there appears to be a transition of dominant populations across a pH gradient (Chen et al., 2013). This transition may be similar to what is experienced during phytostabilization when acidity in mine tailings is often neutralized by a soil amendment to promote vegetation growth. Chen et al. (2014) found that during the moderate pH stage (3–5) of pyrite oxidation, genera with an average relative abundance above 5% included *Tumebacillus*, *Alicyclobacillus*, *Bacillus*, *Acidithiobacillus*, and *Leptospirillum*. Dominant genera in the acidic stage (pH < 3) were *Ferroplasma*, *Sulfobacillus*, and *Leptospirillum*. Intriguingly, several of the genera that were found to naturally colonize pyritic material were capable of both organoheterotrophic as well as lithoautotrophic metabolism. Plant growth itself has shown potential to suppress microbial populations thought to be involved in iron and sulfur oxidation and thus prevent acid generation. A study recently quantified *Acidithiobacillus* and *Leptospirillum*, common iron and sulfur oxidizing bacteria found in AMD, using quantitative PCR and determined that vegetative growth during mine tailing revegetation lowered the abundance of these key iron and sulfur oxidizing bacteria (Li et al., 2016b). Valentín-Vargas et al. (2014) and Valentin-Vargas et al. (2018) showed that plant growth in a revegetation mesocosm experiment acted to delay acidification and was associated with a robust presence of plant-growth-promoting heterotrophs and suppression of lithoautotrophic iron and sulfur oxidizers. Across these studies, it appears that plants have the ability to suppress certain populations of lithotrophic microbes and thereby decrease the rate of acid generation making conditions more conducive to plant growth.

The study presented here contrasts with these previous studies by taxonomically evaluating the microbial dynamics initiated when a plant-growth-supporting microbial community from a compost inoculum is incorporated into a mine tailings environment dominated by acidophiles. The goal is to evaluate a compost-assisted phytostabilization strategy for highly acidic pyritic mine tailings by characterizing the microbial community succession during a 6-year field study following a single application of compost. There are two objectives addressed by this research. The first is to characterize the changes in the bacterial and archaeal microbiome over a 6-year period following a single application of compost to highly acidic pyritic mine tailings in the field. The second is to use a controlled microcosm enrichment study to identify the subset of microbial populations that enable the development and maintenance of acidic conditions when reduced iron and sulfur are present. The combined analysis of microbiome development during a 6-year field study and in a controlled microcosm experiment has elucidated bioindicators that mark transitions between plant-growth-supporting and highly acidic environmental conditions. This research will improve the understanding of bacterial and archaeal microbiome development during phytostabilization and identifies potential bioindicators of the acidification process to improve management of mine tailings reclamation efforts.

## Materials and Methods

### Revegetation Field Study

This study focuses on the temporal progression of 12 phytostabilization plots in the Iron King Mine and Humboldt Smelter Superfund (IKMHSS) field study located in the town of Dewey-Humboldt, AZ, USA. Briefly, this previously described (Hayes et al., 2014; Root et al., 2015) field study was initiated in 2010 to investigate whether compost-assisted phytostabilization could be applied to highly acidic barren pyritic mine tailings to initiate sustained plant growth (Gil-Loaiza et al., 2016; Hammond et al., in preparation). The research presented here utilizes a subset of field trial plots consisting of 10, 15, and 20% (w/w) compost amendment-seeded treatments replicated in quadruplicate. Soil sampling took place annually in May/June (2010–2014, 2016) by collecting 1 m deep cores from each treatment plot in triplicate. Cores were stored on ice until processing in the lab (24–48 h after collection), which involved cutting the cores and archiving samples for different analyses. This investigation utilized only the surficial soil layer (0–20 cm) from one of the triplicate soil cores collected from the center of the plot. Archived samples for DNA extraction were stored at −80°C. Archived sample aliquots for chemical measurements of pH, electrical conductivity (EC), and total carbon (TC) were prepared by freeze-drying to remove moisture. pH and EC measurements were conducted according to EPA method 9045D by creating a 1:1 dry sample weight to milliQ water paste, centrifuging solids, and decanting the supernatant, which was measured for pH and EC using calibrated probes (US EPA, 2004; Hammond et al., in preparation). Total carbon was measured on ground samples using elemental combustion (Costech 4010 CHNS-O, Costech Analytical Technologies Inc., Valencia, CA, USA). Total carbon was assumed to be a proxy for organic carbon, as previously reported values for IKMHSS samples had low inorganic carbon content primarily due to acidic conditions preventing the accumulation of carbonates (Gil-Loaiza et al., 2016). Canopy cover surveys were conducted annually in September/October to correspond with the end of the growing season by a quadrant and transect method (Gil-Loaiza et al., 2016).

### Microcosm Enrichment Cultures

Enrichment of microbial communities involved in acidification through iron and sulfur oxidation was conducted in artificial soil microcosms (Supplementary Figure S1) to simulate the broad range of microniches that exist between soil aggregates (Ellis, 2004). Reduced forms of iron (ferrous sulfate) and sulfur (tetrathionate) were supplied to enrich for microbial communities involved in: (1) iron oxidation (FeO), (2) sulfur oxidation (SO), or (3) concurrent iron and sulfur oxidation (FeSO). Oxidation of reduced sulfur and iron compounds are both directly capable of generating acidity. The reduced sulfur (tetrathionate) can be fully oxidized to sulfate creating free hydrogen ions through a variety of sulfur intermediates. Depending on environmental conditions, including biotic oxidation/reduction activity, intermediate sulfur compounds can also be reduced to form elemental sulfur. The ferrous iron can produce acidity through oxidation to ferric oxyhydroxide.

Two microcosm experiments were performed. The first examined differences in the acidification rate between abiotic and biotic enrichment cultures. Unbuffered moderately acidic pH conditions, with an initial target pH between 4 and 5, were established and pH was not manipulated over the length of the experiment. A second experiment with two pH conditions, buffered moderately acidic and unbuffered highly acidic, was performed to determine if microbial communities enriched from the respective media would be different. The buffered moderately acidic pH condition had an initial target pH of 4–5 and acidity generated during the experiment was neutralized through weekly additions of calcium carbonate. The unbuffered highly acidic pH condition had an initial target pH of 2–3 and the pH was not manipulated after the start of the experiment. The microcosm experiments were sampled every 2 weeks to measure pH and extract DNA for microbial analysis. Buffered moderately acidic cultures had pH measured weekly. Detailed protocols for the creation of the artificial soil matrix and supplied medium, as well as microcosm sampling are described in the Supplementary Material.

### DNA Extraction, Sequencing and Sequence Processing

Field and microcosm sample DNA was extracted from a 0.5 g subsample with the FastDNA SPIN Kit for Soil (MP Biomedicals, Solon OH, USA) with modifications made to the standard FastDNA SPIN KIT for Soil manufacturer’s protocol described previously (Valentín-Vargas et al., 2014). Due to high concentrations of heavy metals in the field samples, all field samples were pre-washed in a 1:1 (w/v) mixture of sample to filter sterilized (0.22 μm) EDTA solution (1 mol L^−1^ EDTA, 100 mmol L^−1^ disodium phosphate, 100 mmol L^−1^ Tris–HCL; titrated to pH 8.2) immediately prior to DNA extraction (Li et al., 2016a). During DNA extraction, blanks that contained no tailings sample were extracted alongside each batch of samples for quality control and subsequently sequenced.

Purified DNA was sent to the University of Arizona Genetics Core (Tucson, AZ, USA) for sequencing on the Illumina MiSeq platform (Illumina, CA, USA; Caporaso et al., 2012). DNA paired-end sequencing was performed on the bacterial and archaeal 16 s rRNA gene V4 hypervariable region achieving a final sequence length of 250 base pairs. The protocol for sequencing procedures is described by Caporaso et al. (2012) with modifications by Laubitz et al. (2016). Samples were sequenced as part of three separate runs: (1) field samples, (2) microcosm samples and the inoculum for the buffered moderately acidic microcosms, and (3) the inoculum for the highly acidic microcosms. A total of 10,500,840 raw sequences from all samples were generated with a median length of 253 base pairs across 149 field and microcosm samples, as well as 21 extraction blanks.

Sequences were processed in QIIME version 1.9.1 (Caporaso et al., 2010). Samples were quality filtered and overlapping ends were joined with a minimum overlap of 20 base pairs. Operational taxonomic units (OTU) were assigned with a 97% similarity threshold using QIIME’s UCLUST-based open-reference OTU picking. Chimeras were filtered out and taxonomy was assigned using the RDP classifier with an 80% confidence threshold using the Greengenes database (Version 13.8, McDonald et al., 2012). It was observed that the Greengenes database incorrectly classified the archaea genera *Ferroplasma* and *Thermogymnomonas* in the family *Picrophilaceae*. Therefore, OTUs classified by the Greengenes database at the genera level to *Ferroplasma* were manually assigned to the family *Ferroplasmaceae* and the genera *Thermogymnomonas* was not assigned a family in accordance with nomenclature standards (Itoh et al., 2007; Golyshina, 2011).

After taxonomic classification, any OTU that was present in the extraction blank was removed from all samples in the extraction set for the associated blank. After sequence and extraction blank filtering a total of 8,297,091 quality sequences remained for an average of 55,685 reads per sample. Any sample that had less than 10,000 reads was removed (12 from the microcosm sample set, 2 from the field study). Samples were then rarefied to 10,000 reads each and subsequent alpha and beta diversity analysis as well as taxonomy summary information was conducted on the rarefied subset using the QIIME software package. Beta diversity analysis was limited to the weighted UniFrac metric (Lozupone and Knight, 2005).

### X-ray Diffraction

A subset of the collected field samples consisting of the 2010, 2012, and 2014 sample years were analyzed by X-ray diffraction (XRD) to quantify the acid generating potential. The crystalline fraction of the surface tailings was determined by Rietveld refinement of Synchrotron transmission powder X-ray diffraction (ST-XRD) as described previously (Hayes et al., 2014; Gil-Loaiza et al., 2016). XRD Laue patterns were collected at Stanford Synchrotron Radiation Lightsource (SSRL) on beamline 11-3 operating at a fixed wavelength of 0.9764 Å in transmission mode using a CCD detector (3,072 × 3,072 pixels). Laue ring images were calibrated with LaB6 and converted to 2θ diffractograms using beamline software. Wavelength conversion from synchrotron to Cu Kα, peak identification, and quantitative analysis of crystalline phases was determined by Rietveld refinement using X’Pert HighScore Plus (PANalytical, version 2.2.3).

The pyrite fraction (% pyt) in the tailings was used to calculate the acid generating potential (AGP), where AGP = % pyt × 16.7; as kg acid equivalents per metric ton tailings expressed as mass equivalents of CaCO_3_ neutralizing capacity of 2 moles CaCO_3_ (MW = 2 × 100.087) to 1 mole of FeS_2_ (MW = 119.975) to kg CaCO_3_ equivalent per ton of material (Parker and Robertson, 1999).

### Statistical Analysis

Statistics were calculated using the JMP 12.0 software package. Statistical significance was determined at *p* < 0.01. Field samples were clustered by Unweighted Pair Group Method with Arithmetic Mean (UPGMA) using the weighted UniFrac beta diversity metric based on the microbial community composition and visualized with TreeGraph 2 (Stöver and Müller, 2010). Comparison between means of field geochemical properties was evaluated with ANOVA and Tukey’s HSD *post hoc* test for means comparison. Comparison between pH values of inoculated and sterile enrichment cultures was conducted through a one-sided student *t*-test. PCoA graphs of enrichment culture and field study samples were generated from the weighted UniFrac distance metric. Statistical significance of sample groups in the enrichment culture and field samples were conducted using ANOSIM with 999 permutations in the QIIME software package. Composited enrichment culture treatments for taxonomic analysis were calculated by determining the median value of OTU reads across the treatment and pH condition replicates and time points. The median values were renormalized with the relative abundance from the median composited values summing to 1. Statistics of the linear regressions between the relative abundance of subset populations in the field microbial communities and pH levels in the field were computed using ANOVA.

## Results

### Development of Field Microbiome and Geochemical Characteristics

**Figure 1:**
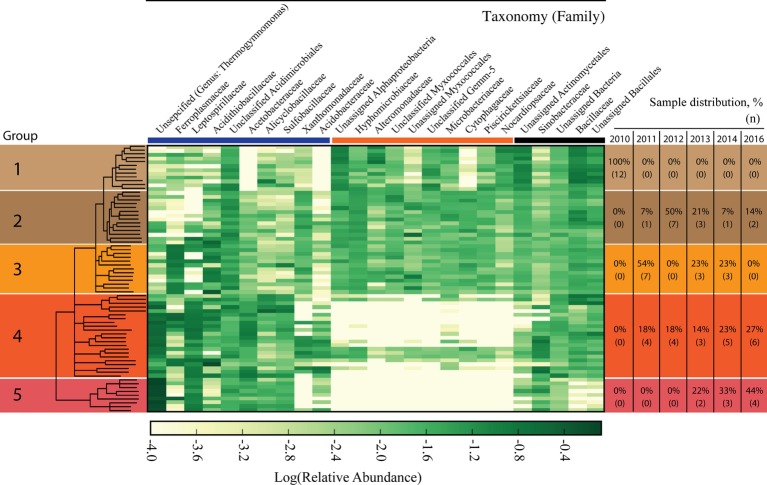
Heat map of microbial families identified in the field study samples. Columns represent family level taxonomy, where families with less than 1% of total relative abundance were removed. Rows represent field samples that have been sorted according to the UPGMA cluster tree generated at the OTU level. The tree is presented in the left column and differentiated into five significantly different Groups (ANOSIM, *p* < 0.01, *R* = 0.65). Nodes with a support value less than 0.5 were collapsed. Relative abundance values have been log transformed and any class that had zero sequence reads has been manually set to −4.0. Darker colors represent more abundant families and whiter colors are less abundant families. The right hand column denotes the distribution of when samples in each of the five Groups were collected. The percentages represent the samples that were collected in each year for a given Group; the number of samples (*n*) present in a year for the Group is given in parenthesis below. The bolded colored lines between the top of the heatmap and family names break the taxonomic families into three sections; the blue line denotes families that are associated with acid mine drainage, orange line denotes families that are strongly selected against in Groups 4 and 5, and the black line denotes other families.

The microbiome, as characterized by bacterial and archaeal populations, in IKMHSS field samples showed a marked transition over the 6-year timeframe examined (2010–2016). The field samples were sorted according to similarity through UPGMA clustering and differentiated into five significant (ANOSIM, *p* < 0.01, *R* = 0.65) Groups (Supplementary Figure S2). These Groups generally followed a temporal pattern across the length of the study (Figure 1, right side). For example, all Group 1 samples were collected in 2010 while Group 5 samples were collected in 2013 (22%), 2014 (33%), and 2016 (44%).

**Figure 2:**
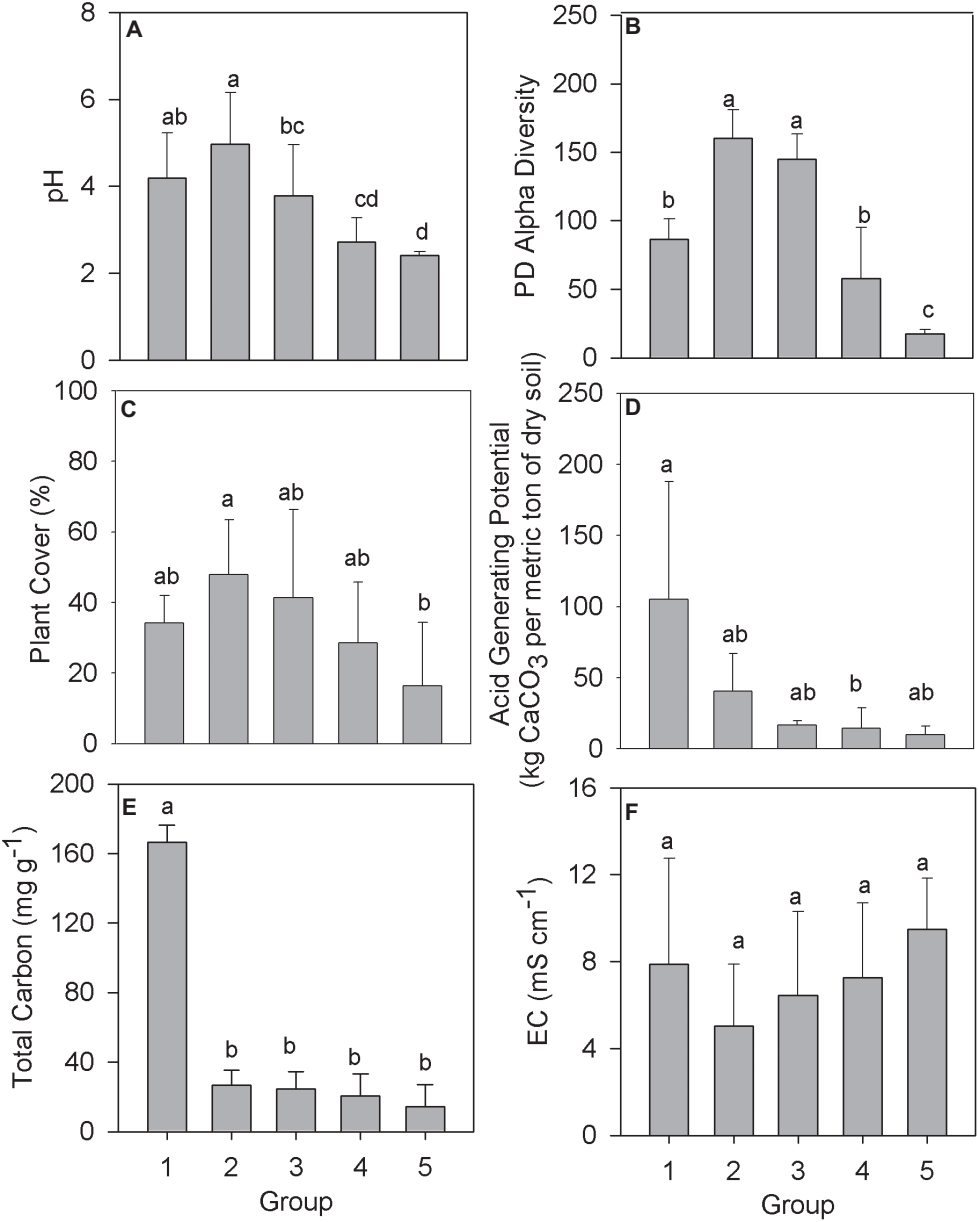
Biogeochemical data from the IKMHSS field study samples as a function of the five field study sample Groups (Figure 1, Supplementary Figure S2). **(A)** pH; **(B)** Alpha diversity; **(C)** % Plant cover; **(D)** Acid generating potential; **(E)** Total carbon; and **(F)** Electrical conductivity. Different letters indicate mean values that are significantly different (*p* < 0.01) across each group. Error bars indicate one standard deviation.

The relationship between community structure and selected environmental parameters in each Group was examined using the averaged biogeochemical properties from samples in that Group (Figure 2). The pH in samples from Group 1 averaged 4.19 (Figure 2A), presumably a result of the compost addition to the tailings, which had an initial pH between 2 and 3. The pH remained above 3 across Groups 2 and 3 before declining in Groups 4 and 5 to a pH below 3. Alpha diversity and plant cover followed a similar trend with increased or similar values among Groups 2 and 3 over Group 1, before declining in value across Groups 4 and 5 (Figures 2B,C). The average acid generating potential (AGP) was highest in samples making up Group 1, with AGP trending downward moving from Group 2 to 5 (Figure 2D). Total soil carbon in samples from Group 1 averaged 166.5 mg g^−1^, significantly higher than every other group (Figure 2E). The consistent residual total carbon values across Groups 2–5, while significantly lower than Group 1, are all higher than the unamended mine tailings, which were below detection limits as reported by (Gil-Loaiza et al., 2016). Finally, electrical conductivity, a proxy measurement of soil salinity, did not differ significantly across any of the denoted groups (Figure 2F).

An examination of the most abundant microbial families in the field study reveals three distinct categories. These categories comprise families associated with AMD (Figure 1, under the blue bar), families strongly selected against in Group 4 and 5 (Figure 1 under the orange bar), and families without a contrasting distribution across the groupings (Figure 1, under the black bar). Ten families were associated with the AMD category and the relative abundance of these families shifted dynamically in different ways in response to the decrease in pH associated with Groups 1–5 (Figure 1 under the blue bar and Figure 2A). For example, *Xanthomonadaceae* was found in samples across Groups 1–3 and 11 of the samples that form Group 4, but was only present in one sample in Group 5. *Acidithiobacillaceae* had the highest relative abundance in Groups 2–4, with a lower relative abundance in the most acidic Group 5. *Ferroplasmaceae* had the highest consistent relative abundance in Group 3. Finally, *Thermogymnomonas* and *Leptospirillaceae* had the highest relative abundance in Groups 4 and 5, while not consistently present in Groups 1–3. Three other AMD-associated families, *Alicyclobacillaceae, Acetobacteraceae,* and *Sulfobacillaceae,* were consistently present across the samples in Groups 2–5 and generally increased in relative abundance with more acidic conditions.

The second category of families in the field study was comprised of 10 taxonomic families present in Groups 1–3 that were strongly selected against in the low-pH associated Groups 4 and 5 (Figure 1, under orange bar.). Of these 10 families, six families (Unassigned *Alphaproteobacteria*, *Hyphomicrobiaceae*, *Alteromonadaceae*, Unclassified *Myxococcales*, Unassigned *Myxococcales*, *Piscirickettsiaceae*) classify within the *Proteobacteria* phylum. Two families (*Microbacteriaceae* and *Nocardiopsaceae*) classify within the *Actinobacteria* phylum. Interestingly, there are four samples in the more acidic Group 4 that maintained the presence of the 10 families that were selected against in the other samples among Group 4 and 5, but also had high relative abundance of *Thermogymnomonas* and *Leptospirillaceae*.

The third category consisted of five families that did not show such pronounced variation in distributions across the different Groups (Figure 1 under the black bar).

### Controlled Microcosm Enrichment Culture Experiments

**Figure 3:**
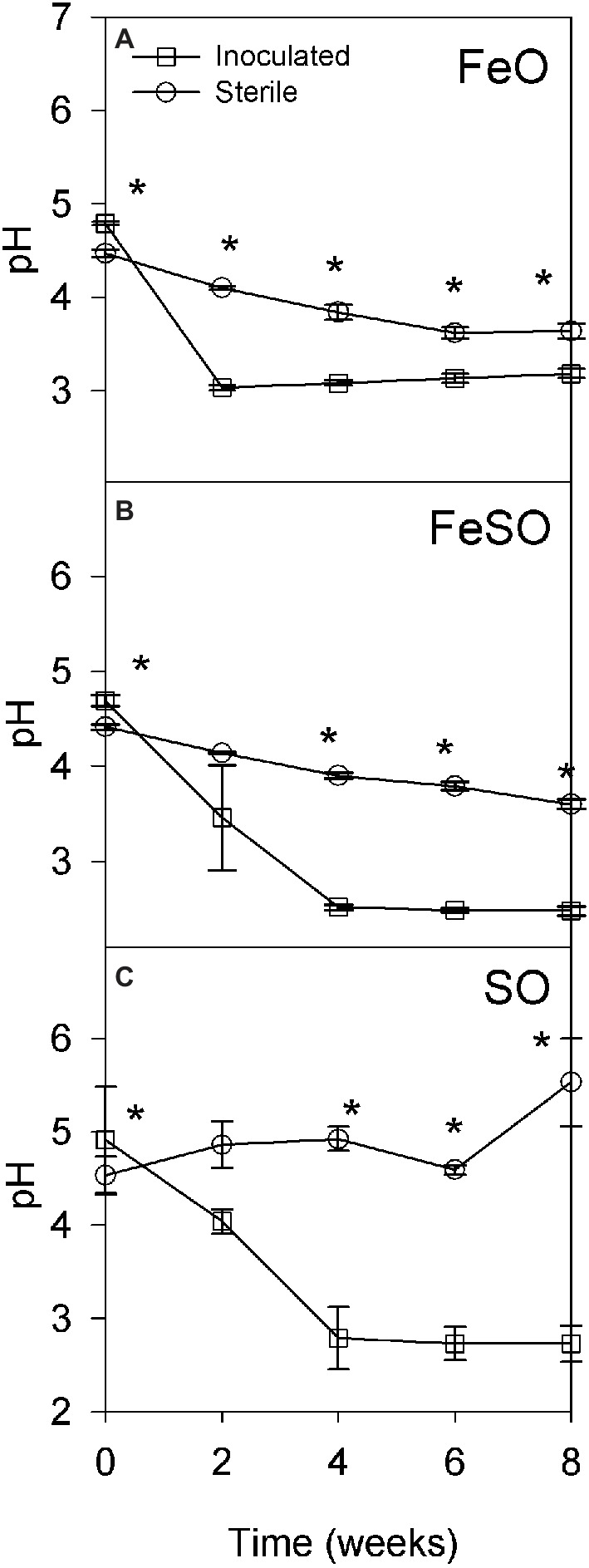
Microcosm experiment to determine pH as a function of time under sterile and inoculated conditions in an unbuffered initially moderately acidic system containing iron and/or sulfur. Three treatments were examined: **(A)** iron oxidation conditions (FeO), **(B)** iron and sulfur oxidation conditions (FeSO), and **(C)** sulfur oxidation conditions (SO). Asterisks (*) denote significantly different (*p* < 0.01) pH values between inoculated and sterile cultures at that time point within the experiment. Error bars indicate one standard deviation.

Two microcosm experiments were performed to identify the subset of microbial populations that enable the development and maintenance of acidic conditions in the presence of reduced iron, reduced sulfur, or a combination of both. The first microcosm experiment was performed in an unbuffered moderately acidic system to determine the relative contribution of abiotic and biotic activity to the development of highly acidic conditions. The initial pH of each treatment FeO, FeSO and SO ranged from 4 to 5. Inoculated treatments acidified to pH ≤ 3.0 after 2, 4, and 4 weeks, respectively (Figures 3A–C). In contrast, sterile FeO and FeSO treatments showed only a slight decrease in pH that was significantly less than in the inoculated treatments (Figures 3A,B). The sterile SO enrichment culture pH increased to 5.5 after 8 weeks (Figure 3C). These results indicate that biological activity plays an important role in increasing acid generation rates under moderately acidic conditions (pH 3–5).

**Figure 4:**
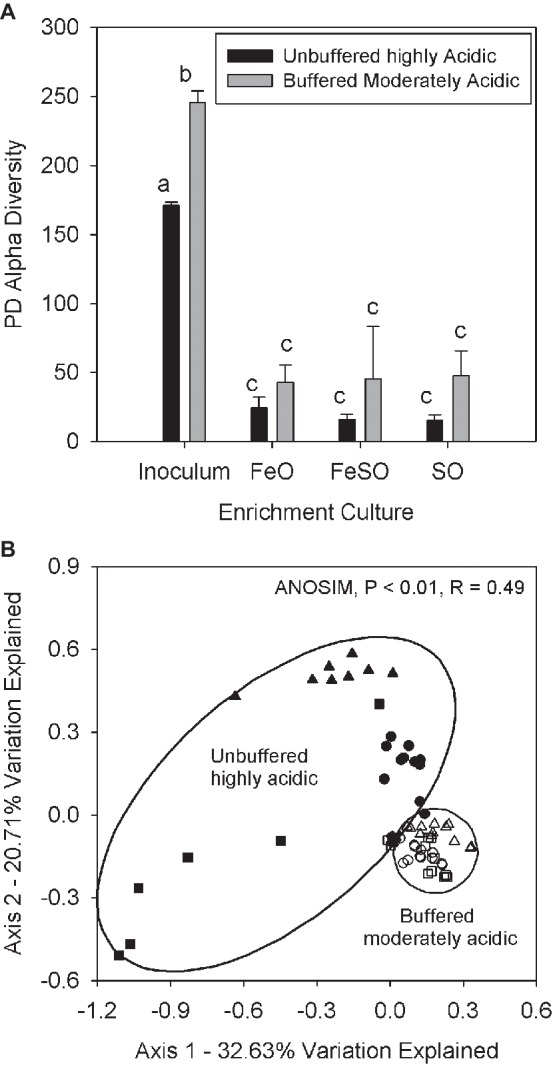
**(A)** Alpha diversity in the FeO, FeSO, and SO microcosm treatments (both the unbuffered highly acidic treatment and the buffered moderately acidic systems) and in the inoculum used for all microcosms. Alpha diversity values were averaged across all replicates and time points. Bars with different letters signify significant differences (*p* < 0.01) between mean values. Error bars indicate one standard deviation. **(B)** PCoA plot of microbial communities from both microcosm systems including all replicates and time points and from the inoculum. Coordinates were determined by UniFrac weighted distance metric. Sample groups are denoted as the inoculum community (x), the buffered moderately acidic system samples are represented by open symbols [FeO (○), FeSO (∆), SO (□)] and the unbuffered highly acidic system samples are represented by solid symbols [FeO (●), FeSO (▲), SO (■)].

The second microcosm experiment was performed with the same treatments to compare a moderately acidic (pH 3–5) system that was buffered to maintain pH with an unbuffered highly acidic (pH 2–3) system. The purpose of this experiment was to determine if changes in the relative abundance of AMD associated families are driven by pH. The buffered moderately acidic system had an initial pH between 4 and 5 and received weekly additions of calcium carbonate to buffer the pH and prevent acidification (Supplementary Figures 3A–C, arrows). In contrast, the unbuffered highly acidic system maintained a pH between 2 and 3 for the duration of the experiment. Alpha diversity measurements in these two systems reveals that each enrichment treatment (FeO, FeSO, SO) had significantly (*p* < 0.01) lower alpha diversity than the inoculum (Figure 4A) in both the buffered moderately acidic and unbuffered highly acidic systems. These results suggest that each treatment (FeO, FeSO, SO) and pH condition selected for a subset of the inoculum microbial community–recall that the inoculum is from the field site.

Gross community composition similarities across the microcosm enrichment experiment were determined through an ordination analysis based on the weighted UniFrac distance metric visualized on a PCoA plot (Figure 4B). The arrangement of the samples within the ordination plot shows a statistically significant (ANOSIM, *p* < 0.01, *R* = 0.49) separation of the buffered moderately acidic and unbuffered highly acidic microbial communities. To examine this further, we identified the taxa that were present with a relative abundance greater than 5% in any microcosm (Table 1). This analysis revealed key taxonomic distinctions between the microbial communities in the buffered moderately acidic and unbuffered highly acidic pH conditions that support the assertion that distinct microbial communities developed. In the buffered moderately acidic pH microcosms, four families (*Alicyclobacillaceae, Bacillaceae, Acetobacteraceae, and Xanthomonadaceae*) were all present at greater than 5% relative abundance in at least two of the three treatments (FeO, FeSO, SO). In contrast, the highly acidic pH microcosms had three families (*Ferroplasmaceae, Sulfobacillaceae, and Leptospirillaceae*) with a relative abundance of greater than 5% in two or more of the treatments. The distribution of *Acidithiobacillaceae*, a well-characterized chemolithoautotroph is intriguing. *Acidithiobacillaceae* was found with similar relative abundance in the FeSO treatment in both the buffered moderately acidic and the unbuffered highly acidic microcosm systems (21.3 and 15.9%, respectively) but was found in low abundance or not at all in the other two treatments (FeO, SO).

**Table 1:** Microbial families with a relative abundance greater than 5% in any enrichment culture treatment.

		Buffered moderately acidic treatments			Unbuffered highly acidic treatments		
Family	Most abundant genus (%)	FeO (%)	FeSO (%)	SO (%)	FeO (%)	FeSO (%)	SO (%)
Alicyclobacillaceae	Alicyclobacillus (48%)	6.9	51.2	5.6	4.8	1.1	0.0
Acetobacteraceae	Acidiphilium (68%)	9.7	1.5	8.1	12.9	0.0	0.0
Xanthomonadaceae	Unassigned (85%)	6.3	7.6	2.6	0.0	0.0	0.0
Bacillaceae	Unassigned (78%)	6.5	5.7	7.2	2.2	0.1	0.9
Ferroplasmaceae	Ferroplasma (100%)	0.0	0.0	0.0	0.3	8.7	84.0
Firmicutes^1^	Sulfobacillus (99%)	0.1	0.1	0.4	10.8	53.2	1.8
Leptospirillaceae	Leptospirillum (100%)	0.0	0.0	0.0	29.9	14.6	3.9
Acidithiobacillaceae	Acidithiobacillus (100%)	0.9	21.3	0.0	0.9	15.9	0.0
Comamonadaceae	Thiomonas (91%)	3.6	0.5	58.9	0.0	0.0	0.0
Unclassified Methylophilales	Unclassified (100%)	40.5	0.0	0.1	0.0	0.0	0.0
Acidimicrobiaceae	Unclassified (100%)	0.0	0.0	0.0	16.5	0.0	0.0
Cumulative Relative Abundance		74.5	87.9	82.9	78.3	93.8	90.6

Relative abundance in each treatment was determined by taking the median value of each OTU across all treatment replicates and time points. OTUs classified at the family level were then summarized and relative abundance values were renormalized. The most abundant genus in each family across all microcosm conditions and treatments is presented in the second column with the percentage of total reads within the family it makes up shown in parentheses. Shaded cells represent a treatment where that family had a relative abundance greater than 5%.

1 Firmicutues represents the phylum in this case.

### Validating Biotic Drivers of Acidification in the IKMHSS Field Microbiome

**Figure 5:**
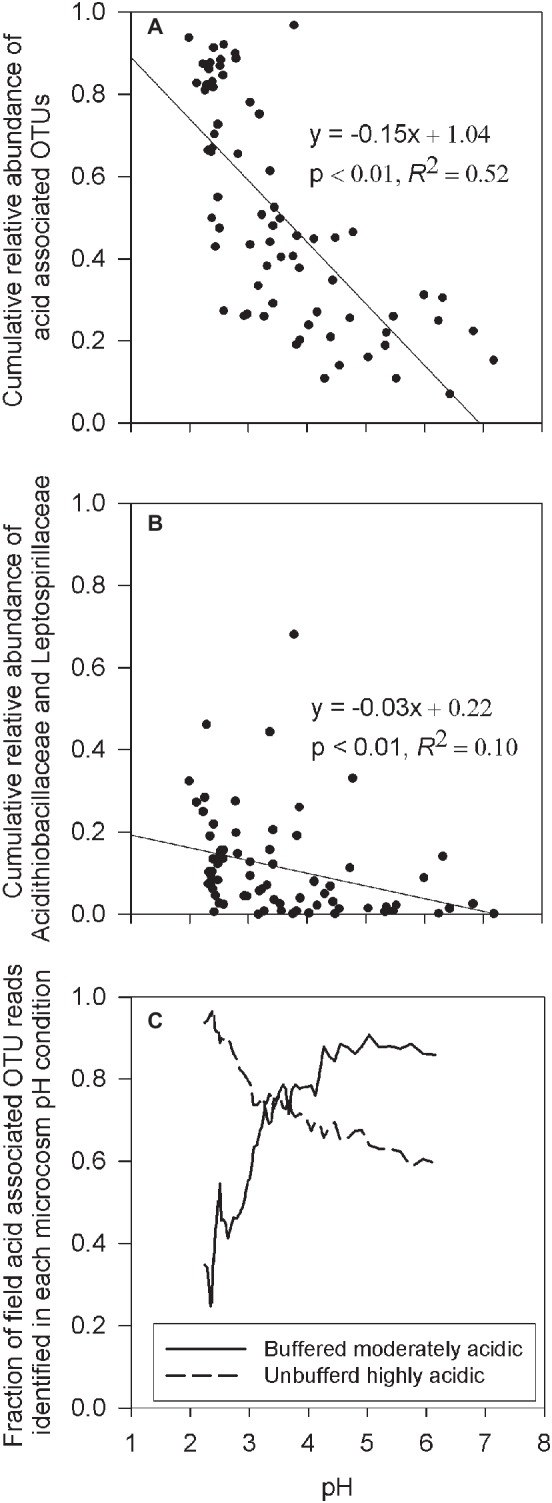
**(A)** Linear regression between the cumulative relative abundance of all acid-associated OTUs (Supplementary Table S1), and the pH of the field sample. **(B)** Linear regression between the cumulative relative abundance of all OTUs classified within the families *Leptospirillaceae* or *Acidithiobacillaceae* and the pH in the field sample. **(C)** Rolling nine sample average of ranked pH data denoting the rolling average fraction of acid-associated OTU reads, in field samples that were identified as OTUs present in either the buffered moderately acidic or unbuffered highly acidic systems, as a function of the field sample pH.

To explore whether the microcosm experiments accurately mimics environmental conditions in the field study, the cumulative relative abundance of OTUs identified in both the microcosm experiments and in the field samples were correlated with field sample pH. For this analysis, all OTUs from the microcosm experiment, with a minimum of five sequence reads across the composited enrichment culture treatments, were defined as an acid-associated OTU (Supplementary Table S1). This resulted in the identification of 398 acid-associated OTUs of which 351 (88.2%) were found in the IKMHSS field samples. Results show a significant (*p* < 0.01) and strong (*R*^2^ = 0.52) linear relationship between the cumulative relative abundance of acid-associated OTUs and field pH (Figure 5A). To understand this result in context, two bacteria well-associated with iron and sulfur oxidation in acid mine drainage, *Leptospirillaceae* and *Acidithiobacillaceae* (Baker and Banfield, 2003), were chosen and similarly correlated with pH. The cumulative relative abundance of OTUs classified in these families (*Acidithiobacillaceae*: 269 OTUs, *Leptospirillaceae*: 142 OTUs) have a significant (*p* < 0.01) but weak (*R*^2^ = 0.10) correlation to field pH (Figure 5B). Taken together, these results suggest that the microcosm experiment did indeed sufficiently mimic the soil environment. Also, these respective correlations suggest that acidic conditions are generated and maintained by a diverse community (Figure 5A) that shifts in response to acidity and not just two major players highly associated with low pH (Figure 5B).

This observation was further confirmed by quantifying the percentage of acid-associated OTUs found in the field samples that were associated with the different microcosm pH conditions. For field samples with pH > 3.5, an average of 84% of the acid-associated OTU reads were OTUs present in buffered moderately acidic microcosms. A smaller average fraction (66%) of the acid-associated OTU reads were identified from OTUs present in the unbuffered highly acidic microcosms (Figure 5C, Supplementary Figure S4). Vice versa, when the pH in the field samples dropped below 3.5, acid-associated OTU reads identified in the field samples were on average 87% in common with the OTUs present in the unbuffered highly acidic microcosms, increasing to an average of 93% in field samples with a pH below 2.5. In contrast, only an average of 49% of the acid-associated OTU reads in field samples with pH < 3.5 were from OTUs identified in buffered moderately acidic microcosms.

## Discussion

This study explored microbiome dynamics in a compost-assisted phytostabilization field study for 6 years following a single application of compost at the beginning of the field trial. Results show a temporal shift over the 6 years from a diverse community capable of supporting plant growth to a community dominated by AMD-associated acidophiles. Results from the complementary microcosm experiments were used to bridge the gap between community taxonomy and function in the field study by empirically associating microbial communities with the functional ability to facilitate the generation or maintenance of acidic conditions. However, evaluation of individual population contributions to acid generation activity becomes nuanced due to poor resolution in assigning definitive taxonomy through amplicon sequencing and the fact that some relevant taxa are associated with both organoheterotrophic and lithoautotrophic metabolisms.

A survey of literature characterizing cultured isolates from families that were abundant in the microcosm experiment (Table 1) suggests that both organoheterotrophic and lithoautotrophic energy metabolisms are present in microbial communities that facilitate acid generation. While lithotrophic activity that catalyzes the oxidation of reduced iron and sulfur is most strongly associated with AMD microbial communities, carbon mineralization through organoheterotrophic activity indirectly promotes environmental conditions conducive to lithotrophic activity and this activity is recognized to play an important role in AMD microbial communities (Baker and Banfield, 2003). Key bacteria and archaea, known to be related to acid generation, are discussed in the following sections in the context of populations identified in this study.

### Bacterial Populations Facilitating Acidification-Putative Lithoautotrophs

Well-characterized obligate lithoautotrophs that directly facilitate the formation of acid in AMD environments were present in both the field and microcosm communities. One example is *Acidithiobacillus ferroxidans*, an aerobic chemolithoautotroph capable of both iron and sulfur oxidation (Ohmura et al., 2002; Valdés et al., 2008). This species has been shown to increase the rate of ferrous iron oxidation below pH 4.5 relative to abiotic conditions, reaching a maximum rate at pH 3 (Meruane and Vargas, 2003). In the present study, all reads corresponding to the family Acidithiobacillaceae were associated with 13 OTUs that were classified to the *Acidithiobacillus* genus level.

A second example is *Leptospirillum*, a well-characterized acidophilic chemolithoautotroph that is highly sensitive to organic carbon and only capable of ferrous iron oxidation (Hallmann et al., 1992; Sand et al., 1992). Recent research has characterized the species *Leptospirillum ferrodiazotrophum* and its ability to fix nitrogen as an important component of AMD microbial communities (Parro and Moreno-Paz, 2003; Tyson et al., 2005). All reads corresponding to the family Leptospirillaceae were associated with seven OTUs that were classified as *Leptospirillum*.

Both of these acidophilic families (Acidithiobacillaceae and Leptospirillaceae) were found at all times throughout the field study (Groups 1–5) and increased in abundance over time. However, as noted earlier, the relative abundance of these families alone was not a good predictor of pH in the field trial.

### Bacterial Populations Facilitating Acidification-Putative Organoheterotrophs

Abundant OTUs from this study that have been linked to organoheterotrophic activity and acidification included four families, the *Acetobacteraceae*, *Alicyclobacillaceae*, *Xanthomonadaceae*, and *Sulfobacillaceae*. For the *Acetobacteraceae,* 66% of reads from the microcosm experiments and 51% of the reads from the field study corresponded to OTUs classified to the genus *Acidiphilium*. *Acidiphilium* is well-characterized as an organoheterotroph in acidic AMD microbial communities (Wichlacz et al., 1986; Chen et al., 2016). Its ability to mineralize organic carbon may play a role in removing products inhibitory to lithotrophic microbial populations (Marchand and Silverstein, 2002; Liu et al., 2011).

In the family *Alicyclobacillaceae*, a reported isolate, *Alicyclobacillus* H1B4, has been shown to be an obligate heterotroph that also has the capacity to oxidize iron (but not sulfur) (Joe et al., 2007). A second reported isolate, *A. ferrooxydans,* is also heterotrophic, but can oxidize both ferrous iron and reduced sulfur compounds (Jiang et al., 2008). In the present study, a total of 62 and 58% of the *Alicyclobacillaceae* reads in the microcosm experiments and field trial respectively, corresponded to *Alicyclobacillus* OTUs. A subset of the *Alicyclobacillus* reads (78% for the microcosm experiment) and (26% for the field trial) corresponded further to *A. ferrooxydans* OTUs.

Similarly, genera within the *Xanthomonadaceae* (found in the buffered moderately acidic FeO and FeSO treatments) have been found in AMD. One species in this family, *Dyella thiooxydans* (not found in this study), has been shown to oxidize sulfur while utilizing several carbon substrates (Lu et al., 2010; Anandham et al., 2011; González-Toril et al., 2011; Chen et al., 2014). A second species that has been found associated with acidic environments is *Metallibacterium scheffleri* strain DKE6, a facultatively anaerobic iron reducer (Ziegler et al., 2013). In this study, a majority of OTUs classified within the Xanthomonadaceae family were not classified to a specific genus.

Finally, a species in the phylum *Firmicutes*, *Sulfobacillus acidophilus,* has been shown to oxidize both iron and sulfur with the capacity to switch between autotrophic and heterotrophic growth (Norris et al., 1996; Karavaiko et al., 2006; Watling et al., 2008). In this study, 13 *Firmicutes* OTUs were identified as *Sulfobacillus* and these OTUs were primarily found under more acidic conditions; in the highly acidic microcosms and in Groups 4 and 5 of the field study.

### Archaeal Populations Facilitating Acidification

The archaea related to acidification in the microcosm and field study samples were comprised of *Ferroplasma* and *Thermogymnomonas*, both of which are associated with highly acidic AMD conditions (Reysenbach and Brileya, 2014). Comparing the potential functional niche of these two genera, *Ferroplasma acidiphilum* has been described as an obligate lithoautotroph (Golyshina et al., 2000). A more recent study has described *F. acidarmanus* as capable of coupling organic carbon oxidation with ferric iron as an alternate electron acceptor under anaerobic conditions (Dopson et al., 2004, 2007). Another study characterized two strains within the *Ferroplasma* that did not grow without a minimal presence of yeast extract and that were also capable of tetrathionate oxidation (Okibe et al., 2003). In contrast, the species *Thermogymnomonas acidicola* has been described as an aerobic chemoheterotroph (Itoh et al., 2007).

There was a distinct shift in the distribution of *Ferroplasmaceae* and *Thermogymnomonas* in the field study. The former was in higher abundance in Group 3 representing higher pH (3–4), while *Thermogymnomonas* was in higher abundance in Groups 4 and 5 at lower pH (2–3) in the field study (Figure 1). This is intriguing since neither genus was present to any great extent in the moderately acidic microcosms but both were present in the highly acidic microcosm (pH 2–3) with *Ferroplasma* dominating. In the highly acidic microcosm, *Ferroplasma* represented 97% of the *Picrophilaceae* reads and *Thermogymnomonas* represented only 3% of reads.

There are similar contrasts in the literature about *Ferroplasma*. It has previously been reported to be abundant in highly acidic AMD microbial communities (Chen et al., 2014). However, Korehi et al. (2014) found that *Ferroplasma* was present during early stages (pH > 5) of natural pyrite oxidation. These results suggest that the behavior of these two archaea may be driven by a combination of pH and other biogeochemical characteristics that has yet to be fully elucidated.

### Microbiome Dynamics in the Field Study

Broadly, results from the IKMHSS field study show an increase in relative abundance of organisms capable of lithotrophic energy metabolisms as the site acidified. The microcosm experiments demonstrate further that it is a co-establishment of potential organoheterotrophic and lithoautotrophic populations that facilitates acidification. The results confirm previous reports of organoheterotrophic organisms in the microbial communities found in highly acidic AMD and early stage moderately acidic pyrite oxidation (Hao et al., 2010; Korehi et al., 2014). These organoheterotrophic and mixotrophic populations may fill a similar niche to that of *Acetobacteraceae* in mineralizing organic compounds that inhibit lithoautotrophic activity (Marchand and Silverstein, 2002; Liu et al., 2011).

Unique to this study are the insights it provides into how the native acidophilic microbial community found in highly acidic mine tailings environment becomes stressed by the addition of an organic amendment (compost) and how this community can eventually recover. Clearly, recovery of the acidophilic community following compost addition requires the participation of organoheterotrophs to mineralize organic carbon to recreate oligotrophic microenvironments that facilitate iron and sulfur oxidation. Such chemoheterotrophic activity may also be important in directly catalyzing the oxidation of pyrite. Hao et al. (2009) showed that co-cultures of chemoheterotrophic and chemolithotrophic bacteria were capable of compromising a phospholipid passivation layer prepared on the surface of pyrite crystals, thereby increasing the rate of pyrite dissolution over monocultures of a lithotrophic bacteria.

We conclude that microorganisms that facilitate acid generation in moderately acidic conditions are key to determining the final outcome in a mine tailings system undergoing compost-assisted phytostabilization. Shifts in the microbial community over the course of this compost-assisted phytostabilization study can be compared to other studies of the progression in microbial communities associated with pyrite oxidation across a pH gradient. Chen et al. (2014) studied natural pyrite oxidation under greenhouse conditions across circumneutral pH to acidic conditions and described a similar set of microbial populations related to *Alicyclobacillaceae*, *Xanthomonadaceae* and *Acetobacteraceae* in more moderately acidic conditions. A second study examined several different mine tailings that exhibited a range of pH (Chen et al., 2013). A 16 s rRNA pyrosequencing analysis of two of the mine tailings, one with higher levels of organic carbon and a pH of 6.4 and the other with lower organic carbon and a pH of 2.4, revealed the following. The site with circumneutral pH conditions had high relative abundance of *Thiobacillus, Legionella, Gemmatimonas, and Sphingomonas,* while the acidic site had high relative abundance of *Ferroplasma*, *Acidithiobacillus*, *Leptospirillum*, *Sulfobacillus* and *Thermogymnomonas*. These results suggest the co-occurrence of *Ferroplasma* and *Thermogymnomonas* in highly acidic conditions and not the distinct shift between the two populations seen in the IKHMSS field study.

While the present study focused on understanding constituent populations of the microbiome that facilitate acidification during phytostabilization, microbial families that support plant growth are equally important to the success of plant establishment. The below ground biomass of plants and the ability to exude organic carbon into the amended soil stimulate organoheterotrophy and suppress lithoautotrophic activity. This process is important to success of above ground biomass production and the presence of plant-growth associated microbial populations actively helps support plant growth, especially in stressed environments (Glick, 2010; Ma et al., 2011). In the taxonomic analysis of the field study microbiome, 8 of the 10 families that exhibited a large decrease in relative abundance under acidifying conditions were either *Proteobacteria* or *Actinobacteria*, taxonomic units strongly associated with natural soils and improved plant growth (Glick, 1995; Spain et al., 2009). One of these families, *Microbacteriaceae*, has related isolates that have been shown to increase the dry weight and length of roots in Rapeseed (*Brassica napus*) grown in lead and copper contaminated soils (Sheng et al., 2008; He et al., 2010). These results suggest that a complete understanding of how best to facilitate phytostabilization will require improved knowledge of how these potential plant-growth-promoting taxonomic families facilitate sustained vegetation growth with a parallel understanding of microbes facilitating acidification. After all, it is the dynamics between these two communities that will decide the success of a phytostabilization attempt.

Therefore, using shifts in energy metabolisms as an indicator for sustained plant growth in reclaimed mine tailings is dependent on the taxa accomplishing this shift in metabolism. Studies of functional activity by transcriptional activity or functional assays would improve the understanding of differences in active energy metabolisms, as well as other functional processes, between plant growth supporting and acid generating microbial communities. Importantly, functional activity studies would be able to elucidate the contribution of mixotrophic populations in microbiome processes and determine how they actively contribute to processes such as iron and sulfur oxidation or carbon cycling across environmental conditions.

## Conclusions

This research demonstrates that microbial activity plays a significant role in acid generation at moderately acidic pH levels where robust plants are still actively growing. The development of microbiomes in mine tailings undergoing phytostabilization at moderately acidic pH conditions appear to be characterized by competing dynamics between acidophilic organoheterotrophic and lithoautotrophic activity that develop acidic conditions and a diverse microbiome that supports plant growth. Taxonomic transitions between these two communities may serve as potential bioindicators of future conditions that either promote or inhibit plant growth. However, taxonomic transitions need to be further validated using physiologically-based studies to tease apart the functional activity between these two competing communities. Efforts to understand the transition between these acidifying and plant-supporting communities would include a more accurate understanding of diagnostic thresholds between a soil microbiome that supports continued plant growth and a microbiome that facilitates the generation of acidity. Combining insights of below ground phytostabilized soil microbiome dynamics with current knowledge of above ground plant growth may better assure sustained long-term vegetation establishment for mine tailing reclamation.

## Data Availability

The 16S rRNA gene datasets for this study can be found in the NCBI Sequence Read Archive database under accession number SRP194659.

## Author Contributions

JH conceived this study, performed microcosm studies and microbial community analysis of microcosm and field samples, statistically analyzed all data, and wrote the manuscript. JG-L and SW were responsible for setting up and maintaining the field study. RR analyzed acid-generating potential of mine tailings samples. JN, JC, and RM contributed to experimental design, data interpretation, and manuscript writing. JC and RM conceived the larger IKHMSS study. All authors reviewed the manuscript.

### Conflict of Interest Statement

The authors declare that the research was conducted in the absence of any commercial or financial relationships that could be construed as a potential conflict of interest.
