# Supplementary material for: Soil Microbiome Dynamics During Pyritic Mine Tailing Phytostabilization: Understanding Microbial Bioindicators of Soil Acidification
Source: Front Microbiol. 2019 Jun 5;10:1211. doi: 10.3389/fmicb.2019.01211 (PMC6593306; doi:10.3389/fmicb.2019.01211)
Supplement: Supplementary file 1 [file Data_Sheet_1.docx]

**Supplemental Information**

**Soil microbiome dynamics during pyritic mine tailing phytostabilization: understanding microbial bioindicators of soil acidification**

John D. Hottenstein, Julie W. Neilson, Juliana Gil-Loaiza, Robert A. Root, Scott A. White, Jon Chorover, Raina M. Maier*

Department of Soil, Water and Environmental Science

The University of Arizona

Tucson, AZ 85721

*Corresponding author

Raina M. Maier

Department of Soil, Water and Environmental Science

University of Arizona

PO Box 210038

Tucson, AZ 85721, USA

[rmaier@ag.arizona.edu](mailto:rmaier@ag.arizona.edu)

**Supplemental Information**

**1.0 Method for initiating the microcosm experiments**

The artificial soil matrix for the microcosm experiment was comprised (w/w) of 50% 20-30 mesh sand (Accusand, UNIMIN Corporation, USA), 35% 50-70 mesh sand, 10% kaolinite, and 5% bentonite clay (Sigma-Aldrich, St. Louis, MO, USA). This mixture was homogenized before dispensing 75g of the combined soil matrix into glass petri dishes (100 x 20 mm). Petri dishes were autoclaved and allowed to cool before designed enrichment medium for microbial iron, iron and sulfur, and sulfur oxidation was supplied based on previously published enrichment media (Johnson et al., 1987; Johnson and McGinness, 1991).

Four solutions were initially prepared with milliQ water. Solution A was a trace element solution consisting with 313 mg L^-1­^ of ZnSO_4_ ▪ 7H_2_O; 31 mg L^-1^ of CuSO_4_ ▪ 5H_2_O, MnSO_4_ ▪ 4H_2_O, and CoSO_4_ ▪ 7H_2_O; and 16 mg L^-1^ of Cr_2_(SO_4_)_3_ ▪ 15H_2_O, Na_2_B_4_O_7_ ▪ 10H_2_O, and NaMoO_4_ ▪ 2H_2_O. Solution B was a basal salt solution with 78.1 g L^-1^ (NH_4_)_2_SO_4_, 31.2 g L^-1^MgSO_4_ ▪ 7H_2_O and 15.6 g L^-1^ Tryptone Soy Broth. Solution C was a ferrous sulfate solution with 97.3 g L^-1^ of FeSO_4_ ▪ 7H_2_O that was acidified to pH 2.0 with sulfuric acid. Solution D was a solution with 30.2 g L^-1^ K_2_S_4_O_6_. Solution A and B were then sterilized by autoclaving, while solution C and D were sterilized by filtration (0.22 μm).1.20 mL of solution A and B was pipetted onto the enrichment culture soil matrix for each culture. 5.30 mL of the ferrous sulfate solution (Solution C) was pipetted onto the FeO and FeSO treatments, while 3.75 mL of the potassium tetrathionate solution (Solution D) was added to the FeSO and SO treatments.

A predetermined amount of sulfuric acid was then pipetted into each enrichment culture to establish the starting pH of either highly acidic or moderately acidic pH conditions. Afterwards, autoclaved milliQ water was used to bring the total volume of liquid added to each culture to 22.5 mL, or 0.30 g g dry soil^-1^ moisture content. Inoculation was accomplished through the incorporation of 0.8g of a surface sample collected from a 15% compost amended plot at the IKMHSS field study in the summer of 2015. Sterile controls for the unbuffered moderately acidic cultures were established by the incorporation of 0.8g of surface mine tailing-compost mixture that had been autoclaved consecutively three times over the period of five days immediately preceding the initiation of the experiment. Enrichment cultures were thoroughly mixed, covered and grown in room temperature conditions, absent of light. The cultures with unbuffered highly acidic and unbuffered moderately acidic conditions were started concurrently, while the cultures with buffered moderately acidic conditions were started approximately six months later. Samples of each inoculum at the time of the experiment initiation were frozen (-80° C) for later DNA extraction and microbial community analysis of the inoculum.

Twice a week, the mass of each enrichment culture was measured and any change in mass not accounted for in sampling was assumed a result of water evaporation. Sterilized milliQ water was added to the enrichment culture to replenish evaporated water on a mass basis. Time 0 pH measurements and soil samples were taken 24 hours after initiation of the experiment. Subsequently, every two weeks from the initiation of the experiment in the highly acidic and unbuffered moderately acidic pH conditions, an approximate 3g soil core was extracted from the culture (Supplementary Figure 1). pH measurements were taken with a 1:1 ratio of approximately 0.5g wet enrichment culture soil matrix and milliQ water. Samples were vortexed for 15 seconds and allowed to settle within a 1.5 mL microcentrifuge tube. pH measurements were then taken on a calibrated pH probe. The remaining soil matrix sample was frozen at -80° C for later DNA extraction. Buffered moderately acidic cultures had pH measurements taken weekly as earlier described. If the culture had acidified, a calculated amount of calcium carbonate was added to raise the pH value. Calcium carbonate was incorporated into the culture by thoroughly mixing. The culture was then allowed to equilibrate for a minimum of one hour before remeasuring **the pH** value.

References:

Johnson, D. B., Macvicar, J. H. M., and Rolfe, S. (1987). A new solid medium for the isolation and enumeration of Thiobacillus ferrooxidans and acidophilic heterotrophic bacteria. J. Microbiol. Methods 7, 9–18. doi: 10.1016/0167- 7012(87)90003-0

Johnson, D. B., and McGinness, S. (1991). A highly effecient and universal solid medium for growing mesophilic and moderately thermophilic, iron-oxidizing, acidophilic bacteria. J. Microbiol. Methods 13, 113–122. doi: 10.1016/0167-7012(91)90011-E

**Supplemental Figures and Tables**


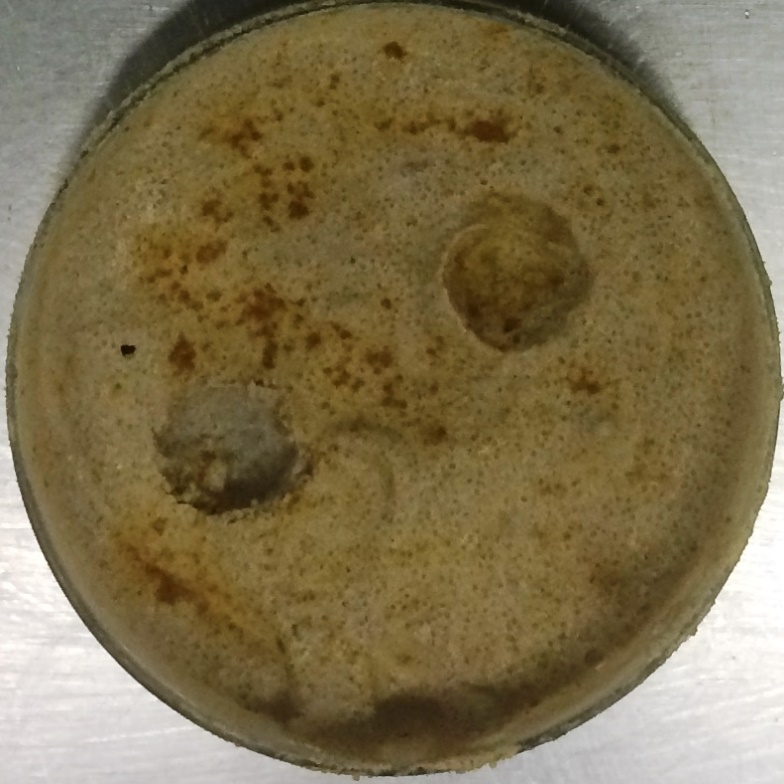


**Supplemental Figure 1.** Picture of the FeO microcosm experiment at 2 weeks. The hole on the bottom left (denoted by arrow) is a “soil core” taken to sample the petri dish for microbial and pH analysis.


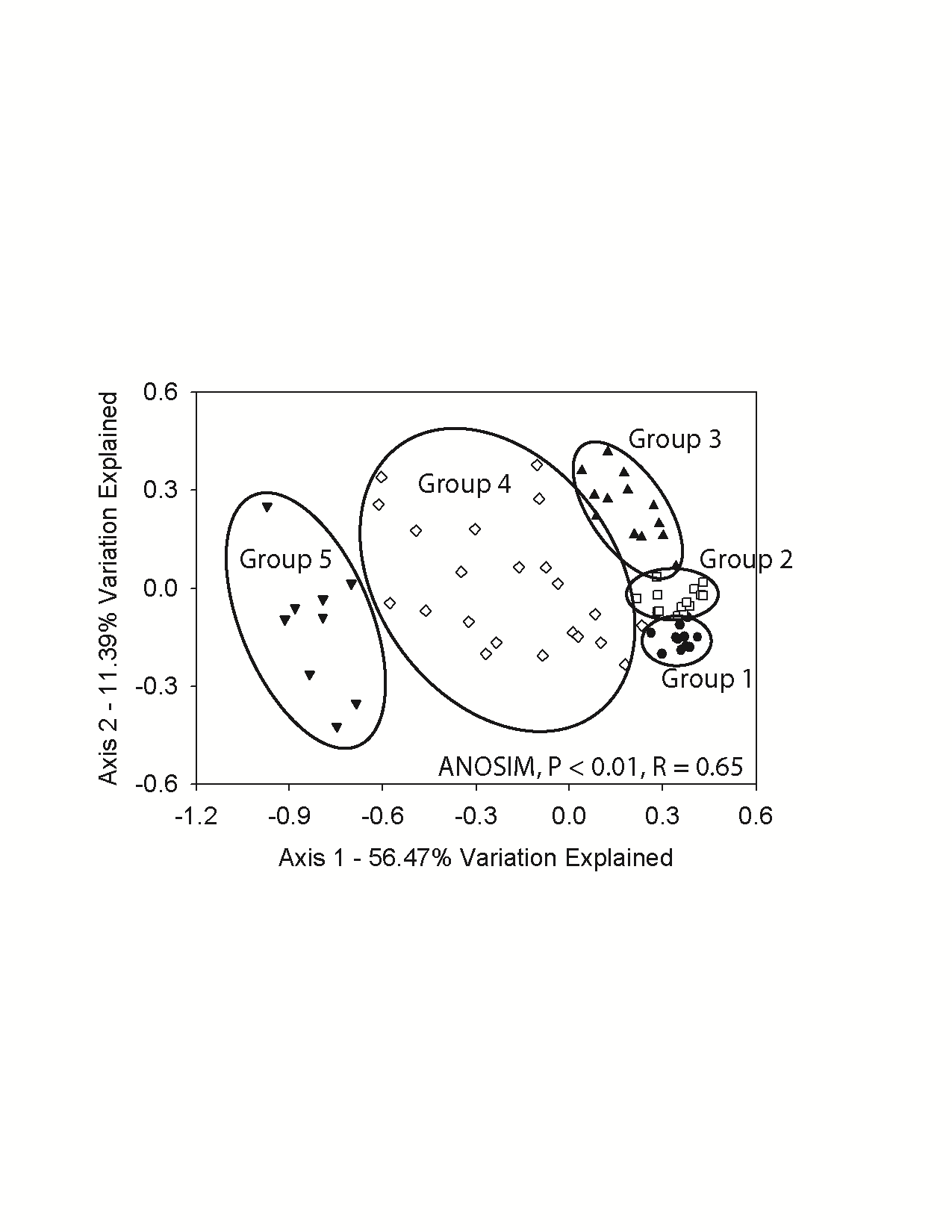


**Supplemental Figure 2.** PCoA plot of field study samples with coordinates determined by UniFrac weighted distance metric and grouped according to the UPGMA cluster tree presented in figure 1. Sample groups are denoted as Group 1 (●), Group 2(□), Group 3 (▲), Group 4 (◇) and Group 5 (▼).

*
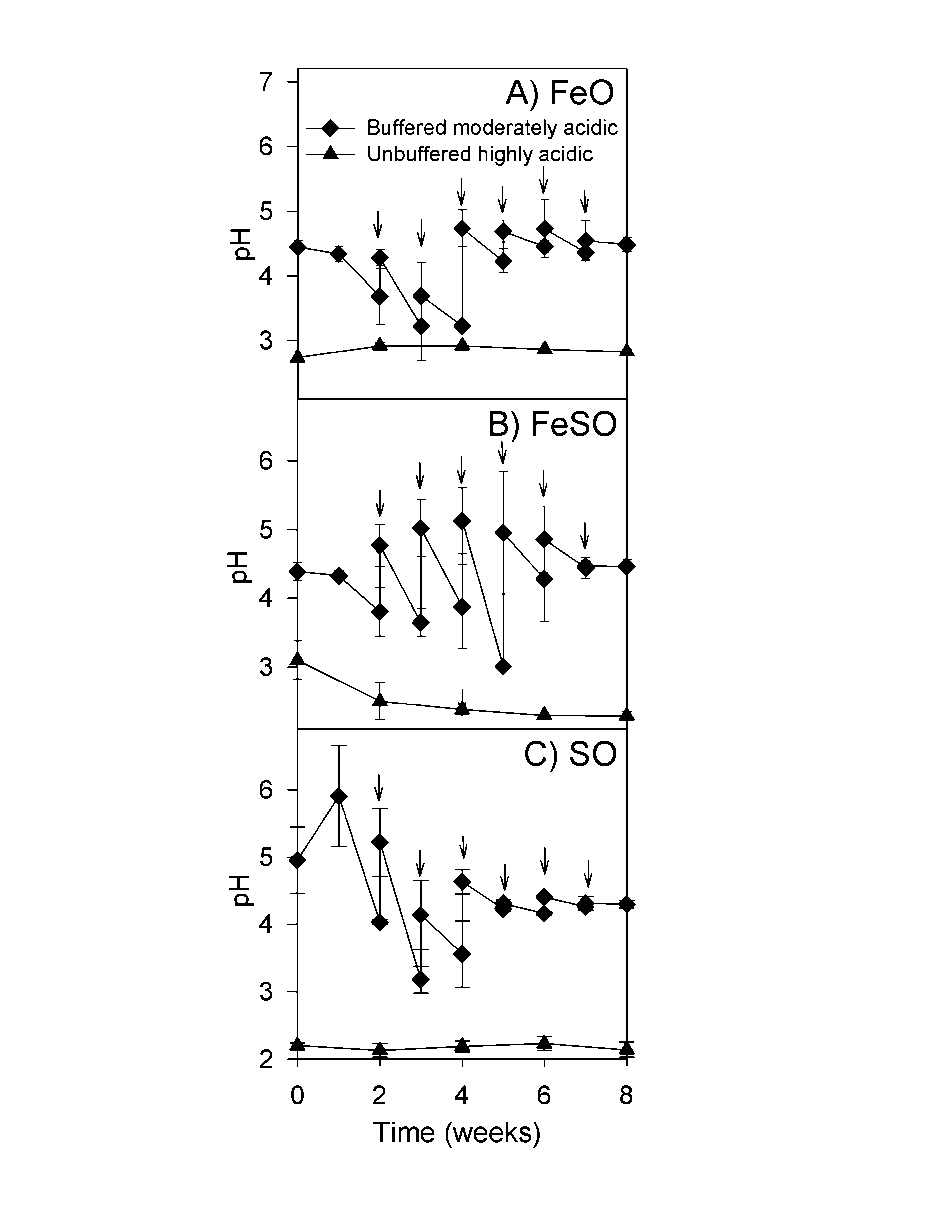
*

**Supplemental Figure 3.** Impact of time on pH in the second microcosm experiment to establish unbuffered highly acidic and buffered moderately acidic pH conditions across (A) iron oxidation, (B) iron and sulfur oxidation, and (C) sulfur oxidation treatments. Same week increases in the pH value within the buffered moderately acidic cultures are due to the incorporation of calcium carbonate (denoted by arrows) to maintain above highly acidic conditions. Error bars indicate one standard deviation.


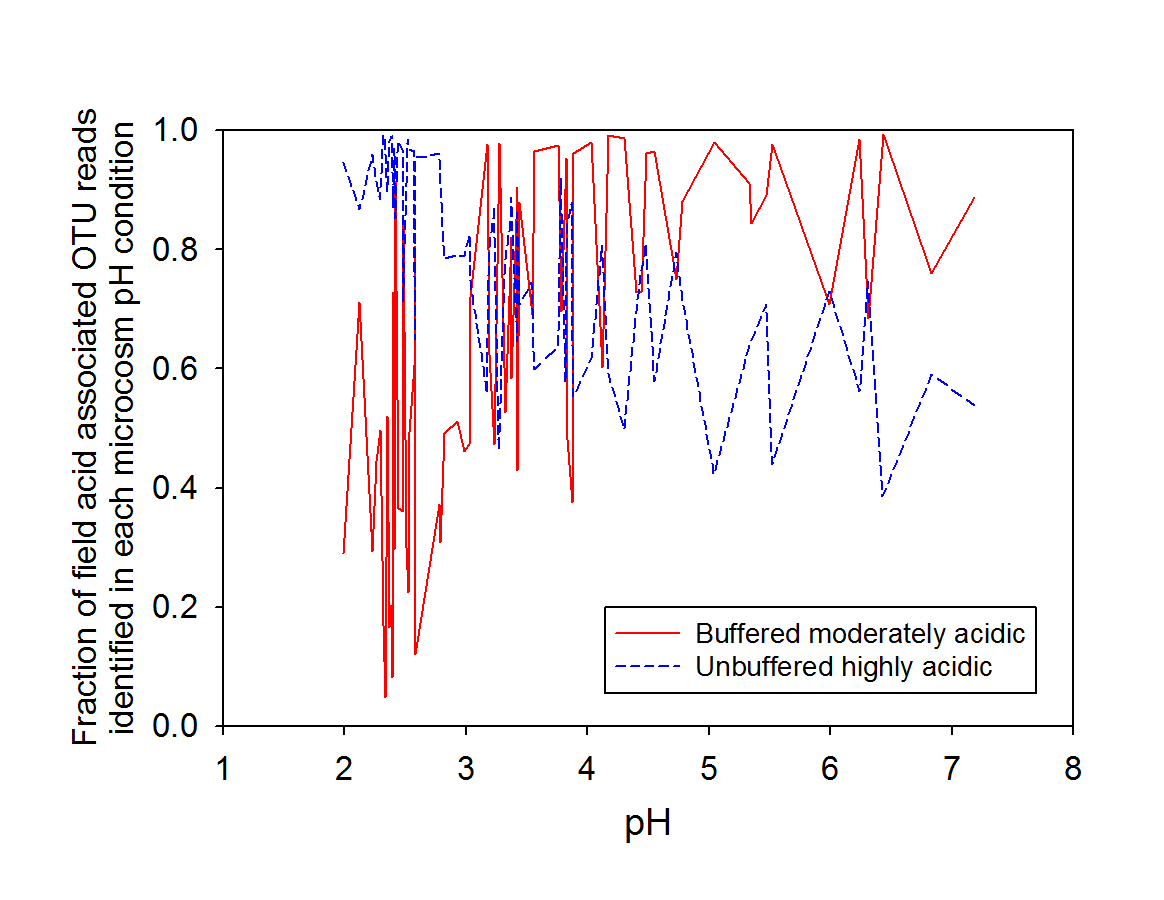


**Supplementary Figure 4.** Raw data of the fraction of acid associated OTU reads in field samples that were identified as OTUs present in either the buffered moderately acidic or unbuffered highly acidic pH conditions as a function of the field sample pH.

**Supplemental Table 1**. The percentage of cumulative sequence reads from each acid associated OTU within the unbuffered highly acidic and buffered moderately acidic enrichment culture pH conditions, as well as within all of the IKMHSS field study samples.

| taxonomy | Unbuffered highly acidic | Buffered moderately acidic | Field samples |
| --- | --- | --- | --- |
| k__Archaea; p__Euryarchaeota; c__Thermoplasmata; o__Thermoplasmatales; f__Picrophilaceae; g__Ferroplasma; s__ | 12.576% | 0.002% | 4.604% |
| k__Archaea; p__Euryarchaeota; c__Thermoplasmata; o__Thermoplasmatales; f__Picrophilaceae; g__Ferroplasma; s__ | 0.035% | 0.000% | 0.012% |
| k__Archaea; p__Euryarchaeota; c__Thermoplasmata; o__Thermoplasmatales; f__Picrophilaceae; g__Ferroplasma; s__ | 0.005% | 0.000% | 0.003% |
| k__Archaea; p__Euryarchaeota; c__Thermoplasmata; o__Thermoplasmatales; f__Picrophilaceae; g__Ferroplasma; s__ | 0.002% | 0.000% | 0.000% |
| k__Archaea; p__Euryarchaeota; c__Thermoplasmata; o__Thermoplasmatales; f__Picrophilaceae; g__Thermogymnomonas; s__ | 0.081% | 0.005% | 5.913% |
| k__Archaea; p__Euryarchaeota; c__Thermoplasmata; o__Thermoplasmatales; f__Picrophilaceae; g__Thermogymnomonas; s__ | 0.161% | 0.001% | 1.316% |
| k__Archaea; p__Euryarchaeota; c__Thermoplasmata; o__Thermoplasmatales; f__Picrophilaceae; g__Thermogymnomonas; s__ | 0.098% | 0.000% | 1.136% |
| k__Archaea; p__Euryarchaeota; c__Thermoplasmata; o__Thermoplasmatales; f__Picrophilaceae; g__Thermogymnomonas; s__ | 0.064% | 0.046% | 1.096% |
| k__Archaea; p__Euryarchaeota; c__Thermoplasmata; o__Thermoplasmatales; f__Picrophilaceae; g__Thermogymnomonas; s__ | 0.021% | 0.003% | 0.879% |
| k__Bacteria | 0.338% | 0.001% | 0.300% |
| k__Bacteria | 0.095% | 0.031% | 0.086% |
| k__Bacteria | 0.084% | 0.020% | 0.065% |
| k__Bacteria | 0.040% | 0.044% | 0.049% |
| k__Bacteria | 0.138% | 0.050% | 0.040% |
| k__Bacteria | 0.033% | 0.018% | 0.031% |
| k__Bacteria | 0.003% | 0.027% | 0.028% |
| k__Bacteria | 0.669% | 0.230% | 0.003% |
| k__Bacteria | 0.000% | 0.013% | 0.000% |
| k__Bacteria | 0.000% | 0.008% | 0.000% |
| k__Bacteria | 0.000% | 0.012% | 0.000% |
| k__Bacteria | 0.000% | 0.005% | 0.000% |
| k__Bacteria; p__Acidobacteria; c__Acidobacteriia; o__Acidobacteriales; f__Acidobacteriaceae; g__; s__ | 0.000% | 0.144% | 0.037% |
| k__Bacteria; p__Acidobacteria; c__Acidobacteriia; o__Acidobacteriales; f__Acidobacteriaceae; g__; s__ | 0.000% | 0.139% | 0.029% |
| k__Bacteria; p__Acidobacteria; c__Acidobacteriia; o__Acidobacteriales; f__Acidobacteriaceae; g__; s__ | 0.000% | 0.282% | 0.010% |
| k__Bacteria; p__Acidobacteria; c__Acidobacteriia; o__Acidobacteriales; f__Acidobacteriaceae; g__; s__ | 0.000% | 0.019% | 0.000% |
| k__Bacteria; p__Acidobacteria; c__Acidobacteriia; o__Acidobacteriales; f__Acidobacteriaceae; g__Acidobacterium; s__ | 0.146% | 0.228% | 0.649% |
| k__Bacteria; p__Actinobacteria; c__Acidimicrobiia; o__Acidimicrobiales | 0.008% | 0.006% | 0.011% |
| k__Bacteria; p__Actinobacteria; c__Acidimicrobiia; o__Acidimicrobiales | 0.000% | 0.041% | 0.003% |
| k__Bacteria; p__Actinobacteria; c__Acidimicrobiia; o__Acidimicrobiales; f__; g__; s__ | 0.886% | 0.068% | 0.317% |
| k__Bacteria; p__Actinobacteria; c__Acidimicrobiia; o__Acidimicrobiales; f__; g__; s__ | 0.229% | 0.029% | 0.163% |
| k__Bacteria; p__Actinobacteria; c__Acidimicrobiia; o__Acidimicrobiales; f__; g__; s__ | 0.104% | 0.001% | 0.160% |
| k__Bacteria; p__Actinobacteria; c__Acidimicrobiia; o__Acidimicrobiales; f__; g__; s__ | 0.043% | 0.001% | 0.156% |
| k__Bacteria; p__Actinobacteria; c__Acidimicrobiia; o__Acidimicrobiales; f__; g__; s__ | 0.070% | 0.030% | 0.132% |
| k__Bacteria; p__Actinobacteria; c__Acidimicrobiia; o__Acidimicrobiales; f__; g__; s__ | 0.278% | 0.112% | 0.096% |
| k__Bacteria; p__Actinobacteria; c__Acidimicrobiia; o__Acidimicrobiales; f__; g__; s__ | 0.081% | 0.077% | 0.032% |
| k__Bacteria; p__Actinobacteria; c__Acidimicrobiia; o__Acidimicrobiales; f__; g__; s__ | 0.022% | 0.013% | 0.031% |
| k__Bacteria; p__Actinobacteria; c__Acidimicrobiia; o__Acidimicrobiales; f__; g__; s__ | 0.019% | 0.001% | 0.021% |
| k__Bacteria; p__Actinobacteria; c__Acidimicrobiia; o__Acidimicrobiales; f__; g__; s__ | 0.005% | 0.001% | 0.013% |
| k__Bacteria; p__Actinobacteria; c__Acidimicrobiia; o__Acidimicrobiales; f__; g__; s__ | 0.018% | 0.006% | 0.006% |
| k__Bacteria; p__Actinobacteria; c__Acidimicrobiia; o__Acidimicrobiales; f__; g__; s__ | 0.000% | 0.007% | 0.004% |
| k__Bacteria; p__Actinobacteria; c__Acidimicrobiia; o__Acidimicrobiales; f__; g__; s__ | 0.003% | 0.001% | 0.002% |
| k__Bacteria; p__Actinobacteria; c__Acidimicrobiia; o__Acidimicrobiales; f__; g__; s__ | 0.025% | 0.002% | 0.001% |
| k__Bacteria; p__Actinobacteria; c__Acidimicrobiia; o__Acidimicrobiales; f__Acidimicrobiaceae; g__; s__ | 7.878% | 0.013% | 0.126% |
| k__Bacteria; p__Actinobacteria; c__Acidimicrobiia; o__Acidimicrobiales; f__Acidimicrobiaceae; g__; s__ | 0.674% | 0.017% | 0.015% |
| k__Bacteria; p__Actinobacteria; c__Actinobacteria; o__Actinomycetales | 0.002% | 0.091% | 0.699% |
| k__Bacteria; p__Actinobacteria; c__Actinobacteria; o__Actinomycetales | 0.000% | 0.221% | 0.186% |
| k__Bacteria; p__Actinobacteria; c__Actinobacteria; o__Actinomycetales | 0.513% | 0.053% | 0.109% |
| k__Bacteria; p__Actinobacteria; c__Actinobacteria; o__Actinomycetales | 0.160% | 0.120% | 0.029% |
| k__Bacteria; p__Actinobacteria; c__Actinobacteria; o__Actinomycetales | 0.000% | 0.042% | 0.003% |
| k__Bacteria; p__Actinobacteria; c__Actinobacteria; o__Actinomycetales | 0.012% | 0.004% | 0.002% |
| k__Bacteria; p__Actinobacteria; c__Actinobacteria; o__Actinomycetales | 0.016% | 0.001% | 0.000% |
| k__Bacteria; p__Actinobacteria; c__Actinobacteria; o__Actinomycetales | 0.015% | 0.007% | 0.000% |
| k__Bacteria; p__Actinobacteria; c__Actinobacteria; o__Actinomycetales; f__; g__; s__ | 0.028% | 0.040% | 0.031% |
| k__Bacteria; p__Actinobacteria; c__Actinobacteria; o__Actinomycetales; f__Cellulomonadaceae; g__Cellulomonas; s__ | 0.000% | 0.671% | 0.037% |
| k__Bacteria; p__Actinobacteria; c__Actinobacteria; o__Actinomycetales; f__Cellulomonadaceae; g__Cellulomonas; s__ | 0.000% | 0.166% | 0.000% |
| k__Bacteria; p__Actinobacteria; c__Actinobacteria; o__Actinomycetales; f__Corynebacteriaceae; g__Corynebacterium; s__ | 0.784% | 0.015% | 0.000% |
| k__Bacteria; p__Actinobacteria; c__Actinobacteria; o__Actinomycetales; f__Frankiaceae; g__; s__ | 0.000% | 0.023% | 0.007% |
| k__Bacteria; p__Actinobacteria; c__Actinobacteria; o__Actinomycetales; f__Microbacteriaceae | 0.002% | 0.015% | 0.364% |
| k__Bacteria; p__Actinobacteria; c__Actinobacteria; o__Actinomycetales; f__Microbacteriaceae | 0.000% | 0.112% | 0.303% |
| k__Bacteria; p__Actinobacteria; c__Actinobacteria; o__Actinomycetales; f__Microbacteriaceae | 0.016% | 0.085% | 0.225% |
| k__Bacteria; p__Actinobacteria; c__Actinobacteria; o__Actinomycetales; f__Microbacteriaceae | 0.000% | 0.063% | 0.028% |
| k__Bacteria; p__Actinobacteria; c__Actinobacteria; o__Actinomycetales; f__Microbacteriaceae | 0.006% | 0.311% | 0.014% |
| k__Bacteria; p__Actinobacteria; c__Actinobacteria; o__Actinomycetales; f__Microbacteriaceae | 0.001% | 0.011% | 0.000% |
| k__Bacteria; p__Actinobacteria; c__Actinobacteria; o__Actinomycetales; f__Microbacteriaceae | 0.000% | 0.007% | 0.000% |
| k__Bacteria; p__Actinobacteria; c__Actinobacteria; o__Actinomycetales; f__Microbacteriaceae; g__Cryocola; s__ | 0.000% | 0.034% | 0.006% |
| k__Bacteria; p__Actinobacteria; c__Actinobacteria; o__Actinomycetales; f__Micrococcaceae | 0.000% | 0.042% | 0.002% |
| k__Bacteria; p__Actinobacteria; c__Actinobacteria; o__Actinomycetales; f__Micrococcaceae; g__Sinomonas; s__ | 0.000% | 0.281% | 0.008% |
| k__Bacteria; p__Actinobacteria; c__Actinobacteria; o__Actinomycetales; f__Mycobacteriaceae; g__Mycobacterium; s__ | 0.026% | 0.114% | 0.148% |
| k__Bacteria; p__Actinobacteria; c__Actinobacteria; o__Actinomycetales; f__Mycobacteriaceae; g__Mycobacterium; s__ | 0.095% | 0.038% | 0.133% |
| k__Bacteria; p__Actinobacteria; c__Actinobacteria; o__Actinomycetales; f__Mycobacteriaceae; g__Mycobacterium; s__ | 0.107% | 0.009% | 0.037% |
| k__Bacteria; p__Actinobacteria; c__Actinobacteria; o__Actinomycetales; f__Pseudonocardiaceae; g__Pseudonocardia | 0.000% | 0.021% | 0.002% |
| k__Bacteria; p__Actinobacteria; c__Actinobacteria; o__Actinomycetales; f__Streptomycetaceae; g__Streptomyces | 0.005% | 0.128% | 0.028% |
| k__Bacteria; p__Actinobacteria; c__Actinobacteria; o__Actinomycetales; f__Thermomonosporaceae; g__Actinomadura; s__vinacea | 0.000% | 0.013% | 0.234% |
| k__Bacteria; p__Actinobacteria; c__Thermoleophilia; o__Gaiellales; f__Gaiellaceae; g__; s__ | 0.000% | 0.041% | 0.000% |
| k__Bacteria; p__Actinobacteria; c__Thermoleophilia; o__Solirubrobacterales | 0.018% | 0.018% | 0.232% |
| k__Bacteria; p__Actinobacteria; c__Thermoleophilia; o__Solirubrobacterales; f__; g__; s__ | 0.000% | 0.261% | 0.053% |
| k__Bacteria; p__Actinobacteria; c__Thermoleophilia; o__Solirubrobacterales; f__Conexibacteraceae; g__; s__ | 0.130% | 0.454% | 0.121% |
| k__Bacteria; p__Actinobacteria; c__Thermoleophilia; o__Solirubrobacterales; f__Conexibacteraceae; g__; s__ | 0.060% | 0.036% | 0.018% |
| k__Bacteria; p__Actinobacteria; c__Thermoleophilia; o__Solirubrobacterales; f__Conexibacteraceae; g__Conexibacter; s__ | 0.081% | 0.017% | 0.053% |
| k__Bacteria; p__Actinobacteria; c__Thermoleophilia; o__Solirubrobacterales; f__Conexibacteraceae; g__Conexibacter; s__ | 0.037% | 0.085% | 0.040% |
| k__Bacteria; p__Actinobacteria; c__Thermoleophilia; o__Solirubrobacterales; f__Conexibacteraceae; g__Conexibacter; s__ | 0.014% | 0.000% | 0.001% |
| k__Bacteria; p__Chloroflexi; c__Thermomicrobia; o__; f__; g__; s__ | 0.000% | 0.064% | 0.000% |
| k__Bacteria; p__Cyanobacteria; c__ML635J-21; o__; f__; g__; s__ | 0.804% | 0.035% | 0.008% |
| k__Bacteria; p__Firmicutes | 0.000% | 0.084% | 0.009% |
| k__Bacteria; p__Firmicutes | 0.026% | 0.014% | 0.006% |
| k__Bacteria; p__Firmicutes; c__Bacilli | 0.001% | 0.011% | 0.006% |
| k__Bacteria; p__Firmicutes; c__Bacilli; o__Bacillales | 2.910% | 1.289% | 1.064% |
| k__Bacteria; p__Firmicutes; c__Bacilli; o__Bacillales | 0.884% | 0.975% | 0.669% |
| k__Bacteria; p__Firmicutes; c__Bacilli; o__Bacillales | 0.094% | 0.321% | 0.359% |
| k__Bacteria; p__Firmicutes; c__Bacilli; o__Bacillales | 0.056% | 0.080% | 0.105% |
| k__Bacteria; p__Firmicutes; c__Bacilli; o__Bacillales | 0.168% | 0.080% | 0.100% |
| k__Bacteria; p__Firmicutes; c__Bacilli; o__Bacillales | 0.182% | 0.110% | 0.078% |
| k__Bacteria; p__Firmicutes; c__Bacilli; o__Bacillales | 0.068% | 0.086% | 0.063% |
| k__Bacteria; p__Firmicutes; c__Bacilli; o__Bacillales | 0.002% | 0.021% | 0.054% |
| k__Bacteria; p__Firmicutes; c__Bacilli; o__Bacillales | 0.037% | 0.042% | 0.053% |
| k__Bacteria; p__Firmicutes; c__Bacilli; o__Bacillales | 0.047% | 0.056% | 0.051% |
| k__Bacteria; p__Firmicutes; c__Bacilli; o__Bacillales | 0.028% | 0.027% | 0.035% |
| k__Bacteria; p__Firmicutes; c__Bacilli; o__Bacillales | 0.029% | 0.046% | 0.035% |
| k__Bacteria; p__Firmicutes; c__Bacilli; o__Bacillales | 0.001% | 0.025% | 0.030% |
| k__Bacteria; p__Firmicutes; c__Bacilli; o__Bacillales | 0.005% | 0.013% | 0.027% |
| k__Bacteria; p__Firmicutes; c__Bacilli; o__Bacillales | 0.023% | 0.035% | 0.022% |
| k__Bacteria; p__Firmicutes; c__Bacilli; o__Bacillales | 0.066% | 0.027% | 0.020% |
| k__Bacteria; p__Firmicutes; c__Bacilli; o__Bacillales | 0.005% | 0.019% | 0.017% |
| k__Bacteria; p__Firmicutes; c__Bacilli; o__Bacillales | 0.022% | 0.032% | 0.013% |
| k__Bacteria; p__Firmicutes; c__Bacilli; o__Bacillales | 0.002% | 0.005% | 0.011% |
| k__Bacteria; p__Firmicutes; c__Bacilli; o__Bacillales | 0.000% | 0.013% | 0.007% |
| k__Bacteria; p__Firmicutes; c__Bacilli; o__Bacillales | 0.004% | 0.022% | 0.002% |
| k__Bacteria; p__Firmicutes; c__Bacilli; o__Bacillales | 0.001% | 0.019% | 0.001% |
| k__Bacteria; p__Firmicutes; c__Bacilli; o__Bacillales | 0.003% | 0.012% | 0.001% |
| k__Bacteria; p__Firmicutes; c__Bacilli; o__Bacillales | 0.002% | 0.034% | 0.001% |
| k__Bacteria; p__Firmicutes; c__Bacilli; o__Bacillales | 0.001% | 0.010% | 0.000% |
| k__Bacteria; p__Firmicutes; c__Bacilli; o__Bacillales | 0.000% | 0.007% | 0.000% |
| k__Bacteria; p__Firmicutes; c__Bacilli; o__Bacillales | 0.001% | 0.013% | 0.000% |
| k__Bacteria; p__Firmicutes; c__Bacilli; o__Bacillales; f__; g__; s__ | 0.431% | 0.399% | 0.404% |
| k__Bacteria; p__Firmicutes; c__Bacilli; o__Bacillales; f__; g__; s__ | 0.021% | 0.028% | 0.047% |
| k__Bacteria; p__Firmicutes; c__Bacilli; o__Bacillales; f__; g__; s__ | 0.000% | 0.008% | 0.013% |
| k__Bacteria; p__Firmicutes; c__Bacilli; o__Bacillales; f__Alicyclobacillaceae | 1.485% | 0.585% | 0.147% |
| k__Bacteria; p__Firmicutes; c__Bacilli; o__Bacillales; f__Alicyclobacillaceae | 0.125% | 0.087% | 0.095% |
| k__Bacteria; p__Firmicutes; c__Bacilli; o__Bacillales; f__Alicyclobacillaceae | 0.096% | 0.044% | 0.050% |
| k__Bacteria; p__Firmicutes; c__Bacilli; o__Bacillales; f__Alicyclobacillaceae | 0.000% | 0.794% | 0.025% |
| k__Bacteria; p__Firmicutes; c__Bacilli; o__Bacillales; f__Alicyclobacillaceae | 0.007% | 0.021% | 0.006% |
| k__Bacteria; p__Firmicutes; c__Bacilli; o__Bacillales; f__Alicyclobacillaceae | 0.000% | 0.024% | 0.001% |
| k__Bacteria; p__Firmicutes; c__Bacilli; o__Bacillales; f__Alicyclobacillaceae | 0.000% | 0.011% | 0.000% |
| k__Bacteria; p__Firmicutes; c__Bacilli; o__Bacillales; f__Alicyclobacillaceae | 0.000% | 0.011% | 0.000% |
| k__Bacteria; p__Firmicutes; c__Bacilli; o__Bacillales; f__Alicyclobacillaceae | 0.000% | 0.007% | 0.000% |
| k__Bacteria; p__Firmicutes; c__Bacilli; o__Bacillales; f__Alicyclobacillaceae; g__; s__ | 0.071% | 2.442% | 0.141% |
| k__Bacteria; p__Firmicutes; c__Bacilli; o__Bacillales; f__Alicyclobacillaceae; g__; s__ | 0.841% | 0.952% | 0.017% |
| k__Bacteria; p__Firmicutes; c__Bacilli; o__Bacillales; f__Alicyclobacillaceae; g__; s__ | 0.000% | 0.112% | 0.006% |
| k__Bacteria; p__Firmicutes; c__Bacilli; o__Bacillales; f__Alicyclobacillaceae; g__; s__ | 0.139% | 0.153% | 0.004% |
| k__Bacteria; p__Firmicutes; c__Bacilli; o__Bacillales; f__Alicyclobacillaceae; g__; s__ | 0.001% | 0.890% | 0.004% |
| k__Bacteria; p__Firmicutes; c__Bacilli; o__Bacillales; f__Alicyclobacillaceae; g__; s__ | 0.000% | 0.015% | 0.001% |
| k__Bacteria; p__Firmicutes; c__Bacilli; o__Bacillales; f__Alicyclobacillaceae; g__; s__ | 0.000% | 0.003% | 0.000% |
| k__Bacteria; p__Firmicutes; c__Bacilli; o__Bacillales; f__Alicyclobacillaceae; g__Alicyclobacillus | 0.099% | 2.760% | 0.484% |
| k__Bacteria; p__Firmicutes; c__Bacilli; o__Bacillales; f__Alicyclobacillaceae; g__Alicyclobacillus | 0.030% | 0.233% | 0.017% |
| k__Bacteria; p__Firmicutes; c__Bacilli; o__Bacillales; f__Alicyclobacillaceae; g__Alicyclobacillus | 0.000% | 0.007% | 0.002% |
| k__Bacteria; p__Firmicutes; c__Bacilli; o__Bacillales; f__Alicyclobacillaceae; g__Alicyclobacillus | 0.000% | 0.005% | 0.002% |
| k__Bacteria; p__Firmicutes; c__Bacilli; o__Bacillales; f__Alicyclobacillaceae; g__Alicyclobacillus | 0.000% | 0.036% | 0.001% |
| k__Bacteria; p__Firmicutes; c__Bacilli; o__Bacillales; f__Alicyclobacillaceae; g__Alicyclobacillus; s__ferripilum | 0.000% | 0.034% | 0.002% |
| k__Bacteria; p__Firmicutes; c__Bacilli; o__Bacillales; f__Alicyclobacillaceae; g__Alicyclobacillus; s__ferripilum | 0.000% | 0.025% | 0.000% |
| k__Bacteria; p__Firmicutes; c__Bacilli; o__Bacillales; f__Alicyclobacillaceae; g__Alicyclobacillus; s__ferrooxydans | 1.531% | 8.865% | 0.170% |
| k__Bacteria; p__Firmicutes; c__Bacilli; o__Bacillales; f__Alicyclobacillaceae; g__Alicyclobacillus; s__ferrooxydans | 0.076% | 0.279% | 0.002% |
| k__Bacteria; p__Firmicutes; c__Bacilli; o__Bacillales; f__Alicyclobacillaceae; g__Alicyclobacillus; s__ferrooxydans | 0.006% | 0.131% | 0.002% |
| k__Bacteria; p__Firmicutes; c__Bacilli; o__Bacillales; f__Alicyclobacillaceae; g__Alicyclobacillus; s__ferrooxydans | 0.001% | 0.010% | 0.001% |
| k__Bacteria; p__Firmicutes; c__Bacilli; o__Bacillales; f__Alicyclobacillaceae; g__Alicyclobacillus; s__ferrooxydans | 0.001% | 0.027% | 0.000% |
| k__Bacteria; p__Firmicutes; c__Bacilli; o__Bacillales; f__Alicyclobacillaceae; g__Alicyclobacillus; s__ferrooxydans | 0.004% | 0.095% | 0.000% |
| k__Bacteria; p__Firmicutes; c__Bacilli; o__Bacillales; f__Alicyclobacillaceae; g__Alicyclobacillus; s__ferrooxydans | 0.002% | 0.039% | 0.000% |
| k__Bacteria; p__Firmicutes; c__Bacilli; o__Bacillales; f__Alicyclobacillaceae; g__Alicyclobacillus; s__ferrooxydans | 0.003% | 0.023% | 0.000% |
| k__Bacteria; p__Firmicutes; c__Bacilli; o__Bacillales; f__Alicyclobacillaceae; g__Alicyclobacillus; s__ferrooxydans | 0.002% | 0.007% | 0.000% |
| k__Bacteria; p__Firmicutes; c__Bacilli; o__Bacillales; f__Alicyclobacillaceae; g__Alicyclobacillus; s__ferrooxydans | 0.001% | 0.019% | 0.000% |
| k__Bacteria; p__Firmicutes; c__Bacilli; o__Bacillales; f__Alicyclobacillaceae; g__Alicyclobacillus; s__ferrooxydans | 0.001% | 0.016% | 0.000% |
| k__Bacteria; p__Firmicutes; c__Bacilli; o__Bacillales; f__Alicyclobacillaceae; g__Alicyclobacillus; s__ferrooxydans | 0.000% | 0.103% | 0.000% |
| k__Bacteria; p__Firmicutes; c__Bacilli; o__Bacillales; f__Alicyclobacillaceae; g__Alicyclobacillus; s__ferrooxydans | 0.000% | 0.005% | 0.000% |
| k__Bacteria; p__Firmicutes; c__Bacilli; o__Bacillales; f__Bacillaceae | 0.676% | 0.858% | 0.721% |
| k__Bacteria; p__Firmicutes; c__Bacilli; o__Bacillales; f__Bacillaceae | 0.056% | 0.141% | 0.271% |
| k__Bacteria; p__Firmicutes; c__Bacilli; o__Bacillales; f__Bacillaceae | 0.146% | 0.179% | 0.236% |
| k__Bacteria; p__Firmicutes; c__Bacilli; o__Bacillales; f__Bacillaceae | 0.074% | 0.158% | 0.162% |
| k__Bacteria; p__Firmicutes; c__Bacilli; o__Bacillales; f__Bacillaceae | 0.132% | 0.179% | 0.149% |
| k__Bacteria; p__Firmicutes; c__Bacilli; o__Bacillales; f__Bacillaceae | 0.102% | 0.150% | 0.114% |
| k__Bacteria; p__Firmicutes; c__Bacilli; o__Bacillales; f__Bacillaceae | 0.024% | 0.119% | 0.113% |
| k__Bacteria; p__Firmicutes; c__Bacilli; o__Bacillales; f__Bacillaceae | 0.008% | 0.057% | 0.079% |
| k__Bacteria; p__Firmicutes; c__Bacilli; o__Bacillales; f__Bacillaceae | 0.034% | 0.056% | 0.073% |
| k__Bacteria; p__Firmicutes; c__Bacilli; o__Bacillales; f__Bacillaceae | 0.019% | 0.081% | 0.068% |
| k__Bacteria; p__Firmicutes; c__Bacilli; o__Bacillales; f__Bacillaceae | 0.000% | 0.018% | 0.062% |
| k__Bacteria; p__Firmicutes; c__Bacilli; o__Bacillales; f__Bacillaceae | 0.000% | 0.018% | 0.061% |
| k__Bacteria; p__Firmicutes; c__Bacilli; o__Bacillales; f__Bacillaceae | 0.102% | 0.022% | 0.061% |
| k__Bacteria; p__Firmicutes; c__Bacilli; o__Bacillales; f__Bacillaceae | 0.038% | 0.056% | 0.050% |
| k__Bacteria; p__Firmicutes; c__Bacilli; o__Bacillales; f__Bacillaceae | 0.003% | 0.011% | 0.047% |
| k__Bacteria; p__Firmicutes; c__Bacilli; o__Bacillales; f__Bacillaceae | 0.028% | 0.018% | 0.039% |
| k__Bacteria; p__Firmicutes; c__Bacilli; o__Bacillales; f__Bacillaceae | 0.000% | 0.008% | 0.033% |
| k__Bacteria; p__Firmicutes; c__Bacilli; o__Bacillales; f__Bacillaceae | 0.008% | 0.029% | 0.026% |
| k__Bacteria; p__Firmicutes; c__Bacilli; o__Bacillales; f__Bacillaceae | 0.007% | 0.020% | 0.026% |
| k__Bacteria; p__Firmicutes; c__Bacilli; o__Bacillales; f__Bacillaceae | 0.012% | 0.018% | 0.026% |
| k__Bacteria; p__Firmicutes; c__Bacilli; o__Bacillales; f__Bacillaceae | 0.006% | 0.019% | 0.026% |
| k__Bacteria; p__Firmicutes; c__Bacilli; o__Bacillales; f__Bacillaceae | 0.070% | 0.042% | 0.023% |
| k__Bacteria; p__Firmicutes; c__Bacilli; o__Bacillales; f__Bacillaceae | 0.000% | 1.285% | 0.023% |
| k__Bacteria; p__Firmicutes; c__Bacilli; o__Bacillales; f__Bacillaceae | 0.003% | 0.018% | 0.018% |
| k__Bacteria; p__Firmicutes; c__Bacilli; o__Bacillales; f__Bacillaceae | 0.001% | 0.006% | 0.015% |
| k__Bacteria; p__Firmicutes; c__Bacilli; o__Bacillales; f__Bacillaceae | 0.009% | 0.014% | 0.014% |
| k__Bacteria; p__Firmicutes; c__Bacilli; o__Bacillales; f__Bacillaceae | 0.010% | 0.013% | 0.014% |
| k__Bacteria; p__Firmicutes; c__Bacilli; o__Bacillales; f__Bacillaceae | 0.002% | 0.010% | 0.012% |
| k__Bacteria; p__Firmicutes; c__Bacilli; o__Bacillales; f__Bacillaceae | 0.005% | 0.027% | 0.012% |
| k__Bacteria; p__Firmicutes; c__Bacilli; o__Bacillales; f__Bacillaceae | 0.013% | 0.010% | 0.009% |
| k__Bacteria; p__Firmicutes; c__Bacilli; o__Bacillales; f__Bacillaceae | 0.010% | 0.012% | 0.009% |
| k__Bacteria; p__Firmicutes; c__Bacilli; o__Bacillales; f__Bacillaceae | 0.003% | 0.010% | 0.009% |
| k__Bacteria; p__Firmicutes; c__Bacilli; o__Bacillales; f__Bacillaceae | 0.001% | 0.049% | 0.004% |
| k__Bacteria; p__Firmicutes; c__Bacilli; o__Bacillales; f__Bacillaceae | 0.000% | 0.107% | 0.001% |
| k__Bacteria; p__Firmicutes; c__Bacilli; o__Bacillales; f__Bacillaceae | 0.000% | 0.013% | 0.001% |
| k__Bacteria; p__Firmicutes; c__Bacilli; o__Bacillales; f__Bacillaceae | 0.000% | 0.003% | 0.000% |
| k__Bacteria; p__Firmicutes; c__Bacilli; o__Bacillales; f__Bacillaceae; g__Bacillus | 0.303% | 0.046% | 0.094% |
| k__Bacteria; p__Firmicutes; c__Bacilli; o__Bacillales; f__Bacillaceae; g__Bacillus | 0.000% | 0.007% | 0.002% |
| k__Bacteria; p__Firmicutes; c__Bacilli; o__Bacillales; f__Bacillaceae; g__Bacillus; s__firmus | 0.031% | 0.154% | 0.244% |
| k__Bacteria; p__Firmicutes; c__Bacilli; o__Bacillales; f__Bacillaceae; g__Lentibacillus | 0.228% | 0.411% | 0.344% |
| k__Bacteria; p__Firmicutes; c__Bacilli; o__Bacillales; f__Bacillaceae; g__Lentibacillus | 0.061% | 0.079% | 0.193% |
| k__Bacteria; p__Firmicutes; c__Bacilli; o__Bacillales; f__Bacillaceae; g__Lentibacillus | 0.005% | 0.013% | 0.016% |
| k__Bacteria; p__Firmicutes; c__Bacilli; o__Bacillales; f__Bacillaceae; g__Virgibacillus | 0.013% | 0.028% | 0.032% |
| k__Bacteria; p__Firmicutes; c__Bacilli; o__Bacillales; f__Bacillaceae; g__Virgibacillus | 0.005% | 0.008% | 0.008% |
| k__Bacteria; p__Firmicutes; c__Bacilli; o__Bacillales; f__Bacillaceae; g__Virgibacillus; s__ | 0.181% | 0.233% | 0.294% |
| k__Bacteria; p__Firmicutes; c__Bacilli; o__Bacillales; f__Bacillaceae; g__Virgibacillus; s__ | 0.030% | 0.051% | 0.111% |
| k__Bacteria; p__Firmicutes; c__Bacilli; o__Bacillales; f__Paenibacillaceae | 0.004% | 0.013% | 0.015% |
| k__Bacteria; p__Firmicutes; c__Bacilli; o__Bacillales; f__Paenibacillaceae; g__; s__ | 0.099% | 0.173% | 0.136% |
| k__Bacteria; p__Firmicutes; c__Bacilli; o__Bacillales; f__Paenibacillaceae; g__; s__ | 0.064% | 0.125% | 0.103% |
| k__Bacteria; p__Firmicutes; c__Bacilli; o__Bacillales; f__Paenibacillaceae; g__; s__ | 0.016% | 0.029% | 0.043% |
| k__Bacteria; p__Firmicutes; c__Bacilli; o__Bacillales; f__Paenibacillaceae; g__; s__ | 0.002% | 0.012% | 0.015% |
| k__Bacteria; p__Firmicutes; c__Bacilli; o__Bacillales; f__Paenibacillaceae; g__Ammoniphilus; s__ | 0.045% | 0.131% | 0.165% |
| k__Bacteria; p__Firmicutes; c__Bacilli; o__Bacillales; f__Paenibacillaceae; g__Ammoniphilus; s__ | 0.021% | 0.073% | 0.080% |
| k__Bacteria; p__Firmicutes; c__Bacilli; o__Bacillales; f__Paenibacillaceae; g__Ammoniphilus; s__ | 0.002% | 0.014% | 0.026% |
| k__Bacteria; p__Firmicutes; c__Bacilli; o__Bacillales; f__Paenibacillaceae; g__Ammoniphilus; s__ | 0.004% | 0.011% | 0.009% |
| k__Bacteria; p__Firmicutes; c__Bacilli; o__Bacillales; f__Planococcaceae; g__Ureibacillus; s__ | 0.000% | 0.020% | 0.040% |
| k__Bacteria; p__Firmicutes; c__Bacilli; o__Bacillales; f__Planococcaceae; g__Ureibacillus; s__ | 0.005% | 0.020% | 0.022% |
| k__Bacteria; p__Firmicutes; c__Bacilli; o__Bacillales; f__Staphylococcaceae; g__Staphylococcus; s__ | 0.040% | 0.000% | 0.000% |
| k__Bacteria; p__Firmicutes; c__Bacilli; o__Bacillales; f__Staphylococcaceae; g__Staphylococcus; s__ | 0.006% | 0.000% | 0.000% |
| k__Bacteria; p__Firmicutes; c__Bacilli; o__Bacillales; f__Thermoactinomycetaceae; g__; s__ | 0.262% | 0.304% | 0.222% |
| k__Bacteria; p__Firmicutes; c__Bacilli; o__Bacillales; f__Thermoactinomycetaceae; g__; s__ | 0.132% | 0.196% | 0.159% |
| k__Bacteria; p__Firmicutes; c__Bacilli; o__Bacillales; f__Thermoactinomycetaceae; g__; s__ | 0.040% | 0.024% | 0.073% |
| k__Bacteria; p__Firmicutes; c__Bacilli; o__Bacillales; f__Thermoactinomycetaceae; g__Planifilum; s__ | 0.502% | 1.037% | 0.364% |
| k__Bacteria; p__Firmicutes; c__Bacilli; o__Bacillales; f__Thermoactinomycetaceae; g__Planifilum; s__ | 0.002% | 0.040% | 0.012% |
| k__Bacteria; p__Firmicutes; c__Bacilli; o__Bacillales; f__Thermoactinomycetaceae; g__Planifilum; s__ | 0.009% | 0.019% | 0.010% |
| k__Bacteria; p__Firmicutes; c__Bacilli; o__Lactobacillales; f__Streptococcaceae; g__Streptococcus; s__ | 0.350% | 0.002% | 0.000% |
| k__Bacteria; p__Firmicutes; c__Bacilli; o__Turicibacterales; f__Turicibacteraceae; g__Turicibacter; s__ | 0.194% | 0.009% | 0.002% |
| k__Bacteria; p__Firmicutes; c__Clostridia | 0.046% | 0.012% | 0.023% |
| k__Bacteria; p__Firmicutes; c__Clostridia | 0.018% | 0.015% | 0.019% |
| k__Bacteria; p__Firmicutes; c__Clostridia | 0.002% | 0.014% | 0.011% |
| k__Bacteria; p__Firmicutes; c__Clostridia; o__; f__; g__; s__ | 0.065% | 0.042% | 0.123% |
| k__Bacteria; p__Firmicutes; c__Clostridia; o__; f__; g__; s__ | 0.165% | 0.096% | 0.119% |
| k__Bacteria; p__Firmicutes; c__Clostridia; o__; f__; g__; s__ | 0.058% | 0.068% | 0.112% |
| k__Bacteria; p__Firmicutes; c__Clostridia; o__; f__; g__; s__ | 0.098% | 0.078% | 0.093% |
| k__Bacteria; p__Firmicutes; c__Clostridia; o__; f__; g__; s__ | 0.033% | 0.040% | 0.035% |
| k__Bacteria; p__Firmicutes; c__Clostridia; o__; f__; g__; s__ | 0.032% | 0.024% | 0.025% |
| k__Bacteria; p__Firmicutes; c__Clostridia; o__; f__; g__; s__ | 0.013% | 0.014% | 0.021% |
| k__Bacteria; p__Firmicutes; c__Clostridia; o__; f__; g__; s__ | 0.010% | 0.017% | 0.021% |
| k__Bacteria; p__Firmicutes; c__Clostridia; o__; f__; g__; s__ | 0.025% | 0.020% | 0.019% |
| k__Bacteria; p__Firmicutes; c__Clostridia; o__; f__; g__; s__ | 0.009% | 0.014% | 0.015% |
| k__Bacteria; p__Firmicutes; c__Clostridia; o__; f__; g__; s__ | 0.019% | 0.005% | 0.011% |
| k__Bacteria; p__Firmicutes; c__Clostridia; o__; f__; g__; s__ | 0.003% | 0.013% | 0.005% |
| k__Bacteria; p__Firmicutes; c__Clostridia; o__Clostridiales; f__[Tissierellaceae]; g__Anaerococcus; s__ | 0.235% | 0.000% | 0.000% |
| k__Bacteria; p__Firmicutes; c__Clostridia; o__Clostridiales; f__[Tissierellaceae]; g__Tepidimicrobium; s__ | 0.000% | 0.028% | 0.018% |
| k__Bacteria; p__Firmicutes; c__Clostridia; o__Clostridiales; f__Clostridiaceae; g__Alkaliphilus; s__ | 0.002% | 0.078% | 0.116% |
| k__Bacteria; p__Firmicutes; c__Clostridia; o__Clostridiales; f__Clostridiaceae; g__Natronincola_Anaerovirgula; s__ | 0.001% | 0.028% | 0.022% |
| k__Bacteria; p__Firmicutes; c__Clostridia; o__Clostridiales; f__Sulfobacillaceae; g__; s__ | 0.002% | 0.041% | 0.004% |
| k__Bacteria; p__Firmicutes; c__Clostridia; o__Clostridiales; f__Sulfobacillaceae; g__Sulfobacillus | 0.876% | 0.001% | 0.087% |
| k__Bacteria; p__Firmicutes; c__Clostridia; o__Clostridiales; f__Sulfobacillaceae; g__Sulfobacillus | 0.614% | 0.001% | 0.058% |
| k__Bacteria; p__Firmicutes; c__Clostridia; o__Clostridiales; f__Sulfobacillaceae; g__Sulfobacillus | 0.100% | 0.000% | 0.000% |
| k__Bacteria; p__Firmicutes; c__Clostridia; o__Clostridiales; f__Sulfobacillaceae; g__Sulfobacillus; s__ | 9.746% | 0.022% | 0.250% |
| k__Bacteria; p__Firmicutes; c__Clostridia; o__Clostridiales; f__Sulfobacillaceae; g__Sulfobacillus; s__ | 1.777% | 0.004% | 0.218% |
| k__Bacteria; p__Firmicutes; c__Clostridia; o__Clostridiales; f__Sulfobacillaceae; g__Sulfobacillus; s__ | 3.992% | 0.064% | 0.146% |
| k__Bacteria; p__Firmicutes; c__Clostridia; o__Clostridiales; f__Sulfobacillaceae; g__Sulfobacillus; s__ | 0.014% | 0.007% | 0.126% |
| k__Bacteria; p__Firmicutes; c__Clostridia; o__Clostridiales; f__Sulfobacillaceae; g__Sulfobacillus; s__ | 0.039% | 0.000% | 0.072% |
| k__Bacteria; p__Firmicutes; c__Clostridia; o__Clostridiales; f__Sulfobacillaceae; g__Sulfobacillus; s__ | 0.400% | 0.068% | 0.071% |
| k__Bacteria; p__Firmicutes; c__Clostridia; o__Clostridiales; f__Sulfobacillaceae; g__Sulfobacillus; s__ | 3.042% | 0.001% | 0.037% |
| k__Bacteria; p__Firmicutes; c__Clostridia; o__Clostridiales; f__Sulfobacillaceae; g__Sulfobacillus; s__ | 0.552% | 0.000% | 0.028% |
| k__Bacteria; p__Firmicutes; c__Clostridia; o__Clostridiales; f__Sulfobacillaceae; g__Sulfobacillus; s__ | 0.032% | 0.001% | 0.001% |
| k__Bacteria; p__Firmicutes; c__Clostridia; o__Clostridiales; f__Sulfobacillaceae; g__Sulfobacillus; s__ | 0.018% | 0.000% | 0.000% |
| k__Bacteria; p__Firmicutes; c__Clostridia; o__Clostridiales; f__Sulfobacillaceae; g__YNPFFP6; s__ | 0.123% | 0.070% | 0.026% |
| k__Bacteria; p__Firmicutes; c__Clostridia; o__Clostridiales; f__Sulfobacillaceae; g__YNPFFP6; s__ | 0.010% | 0.041% | 0.008% |
| k__Bacteria; p__Firmicutes; c__Clostridia; o__MBA08; f__; g__; s__ | 0.030% | 0.082% | 0.080% |
| k__Bacteria; p__Firmicutes; c__Clostridia; o__MBA08; f__; g__; s__ | 0.020% | 0.020% | 0.049% |
| k__Bacteria; p__Firmicutes; c__Clostridia; o__Natranaerobiales; f__Anaerobrancaceae; g__; s__ | 0.006% | 0.009% | 0.018% |
| k__Bacteria; p__Fusobacteria; c__Fusobacteriia; o__Fusobacteriales; f__Fusobacteriaceae; g__Fusobacterium; s__ | 0.007% | 0.000% | 0.000% |
| k__Bacteria; p__Nitrospirae; c__Nitrospira; o__Nitrospirales; f__[Leptospirillaceae]; g__Leptospirillum; s__ | 11.276% | 0.079% | 4.739% |
| k__Bacteria; p__Nitrospirae; c__Nitrospira; o__Nitrospirales; f__[Leptospirillaceae]; g__Leptospirillum; s__ | 0.524% | 0.005% | 0.722% |
| k__Bacteria; p__Nitrospirae; c__Nitrospira; o__Nitrospirales; f__[Leptospirillaceae]; g__Leptospirillum; s__ | 0.130% | 0.000% | 0.063% |
| k__Bacteria; p__Nitrospirae; c__Nitrospira; o__Nitrospirales; f__[Leptospirillaceae]; g__Leptospirillum; s__ | 0.081% | 0.001% | 0.045% |
| k__Bacteria; p__Nitrospirae; c__Nitrospira; o__Nitrospirales; f__[Leptospirillaceae]; g__Leptospirillum; s__ | 0.028% | 0.000% | 0.018% |
| k__Bacteria; p__Nitrospirae; c__Nitrospira; o__Nitrospirales; f__[Leptospirillaceae]; g__Leptospirillum; s__ | 0.038% | 0.001% | 0.018% |
| k__Bacteria; p__Nitrospirae; c__Nitrospira; o__Nitrospirales; f__[Leptospirillaceae]; g__Leptospirillum; s__ | 0.011% | 0.000% | 0.011% |
| k__Bacteria; p__Planctomycetes; c__Planctomycetia; o__Gemmatales; f__Isosphaeraceae | 0.000% | 0.033% | 0.001% |
| k__Bacteria; p__Planctomycetes; c__Planctomycetia; o__Gemmatales; f__Isosphaeraceae; g__; s__ | 0.006% | 0.033% | 0.005% |
| k__Bacteria; p__Planctomycetes; c__Planctomycetia; o__Gemmatales; f__Isosphaeraceae; g__; s__ | 0.000% | 0.028% | 0.000% |
| k__Bacteria; p__Planctomycetes; c__Planctomycetia; o__Pirellulales; f__Pirellulaceae | 0.000% | 0.011% | 0.000% |
| k__Bacteria; p__Proteobacteria | 0.570% | 0.061% | 0.428% |
| k__Bacteria; p__Proteobacteria | 0.748% | 0.000% | 0.023% |
| k__Bacteria; p__Proteobacteria | 0.068% | 0.000% | 0.005% |
| k__Bacteria; p__Proteobacteria | 0.000% | 0.022% | 0.004% |
| k__Bacteria; p__Proteobacteria | 0.000% | 0.018% | 0.000% |
| k__Bacteria; p__Proteobacteria; c__; o__; f__; g__; s__ | 0.000% | 0.041% | 0.009% |
| k__Bacteria; p__Proteobacteria; c__Alphaproteobacteria | 0.000% | 0.016% | 0.001% |
| k__Bacteria; p__Proteobacteria; c__Alphaproteobacteria; o__Caulobacterales; f__Caulobacteraceae; g__Phenylobacterium; s__ | 0.000% | 0.039% | 0.009% |
| k__Bacteria; p__Proteobacteria; c__Alphaproteobacteria; o__Ellin329; f__; g__; s__ | 0.005% | 1.268% | 0.052% |
| k__Bacteria; p__Proteobacteria; c__Alphaproteobacteria; o__Rhizobiales | 0.000% | 0.024% | 0.002% |
| k__Bacteria; p__Proteobacteria; c__Alphaproteobacteria; o__Rhizobiales | 0.000% | 0.052% | 0.001% |
| k__Bacteria; p__Proteobacteria; c__Alphaproteobacteria; o__Rhizobiales; f__Beijerinckiaceae | 0.000% | 0.015% | 0.003% |
| k__Bacteria; p__Proteobacteria; c__Alphaproteobacteria; o__Rhizobiales; f__Bradyrhizobiaceae; g__Bradyrhizobium; s__ | 0.000% | 0.019% | 0.007% |
| k__Bacteria; p__Proteobacteria; c__Alphaproteobacteria; o__Rhizobiales; f__Hyphomicrobiaceae; g__; s__ | 0.000% | 0.041% | 0.083% |
| k__Bacteria; p__Proteobacteria; c__Alphaproteobacteria; o__Rhizobiales; f__Hyphomicrobiaceae; g__Rhodoplanes; s__ | 0.000% | 0.044% | 0.006% |
| k__Bacteria; p__Proteobacteria; c__Alphaproteobacteria; o__Rhizobiales; f__Hyphomicrobiaceae; g__Rhodoplanes; s__ | 0.002% | 0.019% | 0.002% |
| k__Bacteria; p__Proteobacteria; c__Alphaproteobacteria; o__Rhizobiales; f__Methylocystaceae; g__; s__ | 0.000% | 0.055% | 0.001% |
| k__Bacteria; p__Proteobacteria; c__Alphaproteobacteria; o__Rhizobiales; f__Methylocystaceae; g__; s__ | 0.000% | 0.008% | 0.001% |
| k__Bacteria; p__Proteobacteria; c__Alphaproteobacteria; o__Rhodospirillales; f__Acetobacteraceae | 0.034% | 0.000% | 0.454% |
| k__Bacteria; p__Proteobacteria; c__Alphaproteobacteria; o__Rhodospirillales; f__Acetobacteraceae | 0.086% | 0.610% | 0.130% |
| k__Bacteria; p__Proteobacteria; c__Alphaproteobacteria; o__Rhodospirillales; f__Acetobacteraceae | 0.010% | 0.044% | 0.006% |
| k__Bacteria; p__Proteobacteria; c__Alphaproteobacteria; o__Rhodospirillales; f__Acetobacteraceae | 0.000% | 0.023% | 0.003% |
| k__Bacteria; p__Proteobacteria; c__Alphaproteobacteria; o__Rhodospirillales; f__Acetobacteraceae | 0.000% | 0.085% | 0.003% |
| k__Bacteria; p__Proteobacteria; c__Alphaproteobacteria; o__Rhodospirillales; f__Acetobacteraceae | 0.000% | 0.213% | 0.002% |
| k__Bacteria; p__Proteobacteria; c__Alphaproteobacteria; o__Rhodospirillales; f__Acetobacteraceae | 0.000% | 0.044% | 0.001% |
| k__Bacteria; p__Proteobacteria; c__Alphaproteobacteria; o__Rhodospirillales; f__Acetobacteraceae | 0.001% | 0.005% | 0.001% |
| k__Bacteria; p__Proteobacteria; c__Alphaproteobacteria; o__Rhodospirillales; f__Acetobacteraceae; g__; s__ | 1.099% | 0.261% | 0.647% |
| k__Bacteria; p__Proteobacteria; c__Alphaproteobacteria; o__Rhodospirillales; f__Acetobacteraceae; g__; s__ | 0.006% | 0.193% | 0.021% |
| k__Bacteria; p__Proteobacteria; c__Alphaproteobacteria; o__Rhodospirillales; f__Acetobacteraceae; g__; s__ | 0.019% | 0.083% | 0.012% |
| k__Bacteria; p__Proteobacteria; c__Alphaproteobacteria; o__Rhodospirillales; f__Acetobacteraceae; g__; s__ | 0.000% | 0.070% | 0.004% |
| k__Bacteria; p__Proteobacteria; c__Alphaproteobacteria; o__Rhodospirillales; f__Acetobacteraceae; g__; s__ | 0.000% | 0.062% | 0.003% |
| k__Bacteria; p__Proteobacteria; c__Alphaproteobacteria; o__Rhodospirillales; f__Acetobacteraceae; g__; s__ | 0.000% | 0.050% | 0.001% |
| k__Bacteria; p__Proteobacteria; c__Alphaproteobacteria; o__Rhodospirillales; f__Acetobacteraceae; g__; s__ | 0.000% | 0.024% | 0.001% |
| k__Bacteria; p__Proteobacteria; c__Alphaproteobacteria; o__Rhodospirillales; f__Acetobacteraceae; g__; s__ | 0.000% | 0.028% | 0.000% |
| k__Bacteria; p__Proteobacteria; c__Alphaproteobacteria; o__Rhodospirillales; f__Acetobacteraceae; g__Acidiphilium; s__ | 1.539% | 0.844% | 0.528% |
| k__Bacteria; p__Proteobacteria; c__Alphaproteobacteria; o__Rhodospirillales; f__Acetobacteraceae; g__Acidiphilium; s__ | 1.294% | 2.237% | 0.463% |
| k__Bacteria; p__Proteobacteria; c__Alphaproteobacteria; o__Rhodospirillales; f__Acetobacteraceae; g__Acidiphilium; s__ | 0.024% | 0.001% | 0.237% |
| k__Bacteria; p__Proteobacteria; c__Alphaproteobacteria; o__Rhodospirillales; f__Acetobacteraceae; g__Acidiphilium; s__ | 0.048% | 0.007% | 0.093% |
| k__Bacteria; p__Proteobacteria; c__Alphaproteobacteria; o__Rhodospirillales; f__Acetobacteraceae; g__Acidiphilium; s__ | 0.023% | 0.019% | 0.018% |
| k__Bacteria; p__Proteobacteria; c__Alphaproteobacteria; o__Rhodospirillales; f__Acetobacteraceae; g__Acidisoma; s__ | 0.000% | 0.006% | 0.000% |
| k__Bacteria; p__Proteobacteria; c__Alphaproteobacteria; o__Rhodospirillales; f__Acetobacteraceae; g__Acidisoma; s__ | 0.000% | 0.004% | 0.000% |
| k__Bacteria; p__Proteobacteria; c__Alphaproteobacteria; o__Rhodospirillales; f__Acetobacteraceae; g__Acidocella; s__ | 0.026% | 0.030% | 0.020% |
| k__Bacteria; p__Proteobacteria; c__Alphaproteobacteria; o__Rhodospirillales; f__Rhodospirillaceae; g__; s__ | 0.000% | 0.050% | 0.002% |
| k__Bacteria; p__Proteobacteria; c__Alphaproteobacteria; o__Rickettsiales; f__mitochondria | 0.020% | 0.027% | 0.017% |
| k__Bacteria; p__Proteobacteria; c__Betaproteobacteria | 0.000% | 0.054% | 0.013% |
| k__Bacteria; p__Proteobacteria; c__Betaproteobacteria | 0.000% | 1.367% | 0.002% |
| k__Bacteria; p__Proteobacteria; c__Betaproteobacteria | 0.000% | 0.819% | 0.001% |
| k__Bacteria; p__Proteobacteria; c__Betaproteobacteria | 0.000% | 0.049% | 0.001% |
| k__Bacteria; p__Proteobacteria; c__Betaproteobacteria | 0.001% | 0.038% | 0.000% |
| k__Bacteria; p__Proteobacteria; c__Betaproteobacteria | 0.000% | 0.057% | 0.000% |
| k__Bacteria; p__Proteobacteria; c__Betaproteobacteria | 0.000% | 0.109% | 0.000% |
| k__Bacteria; p__Proteobacteria; c__Betaproteobacteria | 0.000% | 0.038% | 0.000% |
| k__Bacteria; p__Proteobacteria; c__Betaproteobacteria | 0.000% | 0.030% | 0.000% |
| k__Bacteria; p__Proteobacteria; c__Betaproteobacteria | 0.000% | 0.018% | 0.000% |
| k__Bacteria; p__Proteobacteria; c__Betaproteobacteria | 0.000% | 0.002% | 0.000% |
| k__Bacteria; p__Proteobacteria; c__Betaproteobacteria; o__Burkholderiales | 0.000% | 1.323% | 0.003% |
| k__Bacteria; p__Proteobacteria; c__Betaproteobacteria; o__Burkholderiales | 0.000% | 0.023% | 0.000% |
| k__Bacteria; p__Proteobacteria; c__Betaproteobacteria; o__Burkholderiales; f__Alcaligenaceae | 0.000% | 0.204% | 0.003% |
| k__Bacteria; p__Proteobacteria; c__Betaproteobacteria; o__Burkholderiales; f__Burkholderiaceae; g__Burkholderia | 0.017% | 0.017% | 0.000% |
| k__Bacteria; p__Proteobacteria; c__Betaproteobacteria; o__Burkholderiales; f__Comamonadaceae | 0.003% | 0.720% | 0.006% |
| k__Bacteria; p__Proteobacteria; c__Betaproteobacteria; o__Burkholderiales; f__Comamonadaceae | 0.000% | 0.008% | 0.000% |
| k__Bacteria; p__Proteobacteria; c__Betaproteobacteria; o__Burkholderiales; f__Comamonadaceae; g__Thiomonas; s__cuprina | 0.000% | 11.066% | 0.019% |
| k__Bacteria; p__Proteobacteria; c__Betaproteobacteria; o__Burkholderiales; f__Comamonadaceae; g__Thiomonas; s__cuprina | 0.000% | 0.453% | 0.000% |
| k__Bacteria; p__Proteobacteria; c__Betaproteobacteria; o__Burkholderiales; f__Comamonadaceae; g__Thiomonas; s__cuprina | 0.000% | 0.025% | 0.000% |
| k__Bacteria; p__Proteobacteria; c__Betaproteobacteria; o__Burkholderiales; f__Comamonadaceae; g__Thiomonas; s__cuprina | 0.000% | 0.005% | 0.000% |
| k__Bacteria; p__Proteobacteria; c__Betaproteobacteria; o__Burkholderiales; f__Comamonadaceae; g__Thiomonas; s__cuprina | 0.000% | 0.446% | 0.000% |
| k__Bacteria; p__Proteobacteria; c__Betaproteobacteria; o__Burkholderiales; f__Comamonadaceae; g__Thiomonas; s__cuprina | 0.000% | 0.059% | 0.000% |
| k__Bacteria; p__Proteobacteria; c__Betaproteobacteria; o__Burkholderiales; f__Comamonadaceae; g__Thiomonas; s__cuprina | 0.000% | 0.028% | 0.000% |
| k__Bacteria; p__Proteobacteria; c__Betaproteobacteria; o__Burkholderiales; f__Comamonadaceae; g__Thiomonas; s__cuprina | 0.000% | 0.009% | 0.000% |
| k__Bacteria; p__Proteobacteria; c__Betaproteobacteria; o__Burkholderiales; f__Comamonadaceae; g__Thiomonas; s__cuprina | 0.000% | 0.007% | 0.000% |
| k__Bacteria; p__Proteobacteria; c__Betaproteobacteria; o__Burkholderiales; f__Comamonadaceae; g__Thiomonas; s__cuprina | 0.000% | 0.007% | 0.000% |
| k__Bacteria; p__Proteobacteria; c__Betaproteobacteria; o__Burkholderiales; f__Comamonadaceae; g__Thiomonas; s__cuprina | 0.000% | 0.006% | 0.000% |
| k__Bacteria; p__Proteobacteria; c__Betaproteobacteria; o__Burkholderiales; f__Comamonadaceae; g__Thiomonas; s__cuprina | 0.000% | 0.006% | 0.000% |
| k__Bacteria; p__Proteobacteria; c__Betaproteobacteria; o__Burkholderiales; f__Oxalobacteraceae; g__Ralstonia; s__ | 0.601% | 0.030% | 0.007% |
| k__Bacteria; p__Proteobacteria; c__Betaproteobacteria; o__Burkholderiales; f__Oxalobacteraceae; g__Ralstonia; s__ | 0.011% | 0.001% | 0.004% |
| k__Bacteria; p__Proteobacteria; c__Betaproteobacteria; o__Burkholderiales; f__Oxalobacteraceae; g__Ralstonia; s__ | 0.013% | 0.000% | 0.000% |
| k__Bacteria; p__Proteobacteria; c__Betaproteobacteria; o__Burkholderiales; f__Oxalobacteraceae; g__Ralstonia; s__ | 0.010% | 0.000% | 0.000% |
| k__Bacteria; p__Proteobacteria; c__Betaproteobacteria; o__Burkholderiales; f__Oxalobacteraceae; g__Ralstonia; s__ | 0.003% | 0.000% | 0.000% |
| k__Bacteria; p__Proteobacteria; c__Betaproteobacteria; o__Hydrogenophilales; f__Hydrogenophilaceae; g__Thiobacillus; s__ | 0.000% | 0.299% | 0.009% |
| k__Bacteria; p__Proteobacteria; c__Betaproteobacteria; o__Methylophilales; f__; g__; s__ | 0.001% | 6.965% | 0.016% |
| k__Bacteria; p__Proteobacteria; c__Betaproteobacteria; o__Methylophilales; f__; g__; s__ | 0.002% | 0.043% | 0.004% |
| k__Bacteria; p__Proteobacteria; c__Betaproteobacteria; o__Methylophilales; f__; g__; s__ | 0.000% | 0.158% | 0.000% |
| k__Bacteria; p__Proteobacteria; c__Betaproteobacteria; o__Methylophilales; f__; g__; s__ | 0.000% | 0.006% | 0.000% |
| k__Bacteria; p__Proteobacteria; c__Betaproteobacteria; o__SBla14; f__; g__; s__ | 0.000% | 0.012% | 0.000% |
| k__Bacteria; p__Proteobacteria; c__Gammaproteobacteria | 0.000% | 0.057% | 0.018% |
| k__Bacteria; p__Proteobacteria; c__Gammaproteobacteria | 0.000% | 0.020% | 0.004% |
| k__Bacteria; p__Proteobacteria; c__Gammaproteobacteria | 0.000% | 0.476% | 0.000% |
| k__Bacteria; p__Proteobacteria; c__Gammaproteobacteria | 0.000% | 0.020% | 0.000% |
| k__Bacteria; p__Proteobacteria; c__Gammaproteobacteria; o__Acidithiobacillales; f__Acidithiobacillaceae; g__Acidithiobacillus | 0.002% | 0.007% | 0.006% |
| k__Bacteria; p__Proteobacteria; c__Gammaproteobacteria; o__Acidithiobacillales; f__Acidithiobacillaceae; g__Acidithiobacillus | 0.033% | 0.011% | 0.002% |
| k__Bacteria; p__Proteobacteria; c__Gammaproteobacteria; o__Acidithiobacillales; f__Acidithiobacillaceae; g__Acidithiobacillus; s__ | 4.266% | 4.991% | 4.215% |
| k__Bacteria; p__Proteobacteria; c__Gammaproteobacteria; o__Acidithiobacillales; f__Acidithiobacillaceae; g__Acidithiobacillus; s__ | 0.921% | 1.727% | 1.049% |
| k__Bacteria; p__Proteobacteria; c__Gammaproteobacteria; o__Acidithiobacillales; f__Acidithiobacillaceae; g__Acidithiobacillus; s__ | 0.069% | 0.110% | 0.081% |
| k__Bacteria; p__Proteobacteria; c__Gammaproteobacteria; o__Acidithiobacillales; f__Acidithiobacillaceae; g__Acidithiobacillus; s__ | 0.072% | 0.128% | 0.074% |
| k__Bacteria; p__Proteobacteria; c__Gammaproteobacteria; o__Acidithiobacillales; f__Acidithiobacillaceae; g__Acidithiobacillus; s__ | 0.010% | 0.038% | 0.028% |
| k__Bacteria; p__Proteobacteria; c__Gammaproteobacteria; o__Acidithiobacillales; f__Acidithiobacillaceae; g__Acidithiobacillus; s__ | 0.009% | 0.017% | 0.008% |
| k__Bacteria; p__Proteobacteria; c__Gammaproteobacteria; o__Acidithiobacillales; f__Acidithiobacillaceae; g__Acidithiobacillus; s__ | 0.000% | 0.046% | 0.007% |
| k__Bacteria; p__Proteobacteria; c__Gammaproteobacteria; o__Acidithiobacillales; f__Acidithiobacillaceae; g__Acidithiobacillus; s__ | 0.011% | 0.013% | 0.006% |
| k__Bacteria; p__Proteobacteria; c__Gammaproteobacteria; o__Acidithiobacillales; f__Acidithiobacillaceae; g__Acidithiobacillus; s__ | 0.006% | 0.080% | 0.001% |
| k__Bacteria; p__Proteobacteria; c__Gammaproteobacteria; o__Acidithiobacillales; f__Acidithiobacillaceae; g__Acidithiobacillus; s__albertensis | 0.013% | 0.013% | 0.009% |
| k__Bacteria; p__Proteobacteria; c__Gammaproteobacteria; o__Acidithiobacillales; f__Acidithiobacillaceae; g__Acidithiobacillus; s__albertensis | 0.007% | 0.007% | 0.008% |
| k__Bacteria; p__Proteobacteria; c__Gammaproteobacteria; o__Xanthomonadales; f__Sinobacteraceae; g__; s__ | 1.004% | 0.107% | 1.438% |
| k__Bacteria; p__Proteobacteria; c__Gammaproteobacteria; o__Xanthomonadales; f__Sinobacteraceae; g__; s__ | 0.207% | 0.054% | 0.211% |
| k__Bacteria; p__Proteobacteria; c__Gammaproteobacteria; o__Xanthomonadales; f__Sinobacteraceae; g__; s__ | 0.096% | 0.029% | 0.093% |
| k__Bacteria; p__Proteobacteria; c__Gammaproteobacteria; o__Xanthomonadales; f__Sinobacteraceae; g__; s__ | 0.000% | 0.385% | 0.069% |
| k__Bacteria; p__Proteobacteria; c__Gammaproteobacteria; o__Xanthomonadales; f__Sinobacteraceae; g__; s__ | 0.006% | 0.130% | 0.031% |
| k__Bacteria; p__Proteobacteria; c__Gammaproteobacteria; o__Xanthomonadales; f__Sinobacteraceae; g__; s__ | 0.026% | 0.021% | 0.021% |
| k__Bacteria; p__Proteobacteria; c__Gammaproteobacteria; o__Xanthomonadales; f__Sinobacteraceae; g__; s__ | 0.014% | 0.004% | 0.012% |
| k__Bacteria; p__Proteobacteria; c__Gammaproteobacteria; o__Xanthomonadales; f__Sinobacteraceae; g__; s__ | 0.000% | 0.102% | 0.008% |
| k__Bacteria; p__Proteobacteria; c__Gammaproteobacteria; o__Xanthomonadales; f__Sinobacteraceae; g__; s__ | 0.003% | 0.049% | 0.005% |
| k__Bacteria; p__Proteobacteria; c__Gammaproteobacteria; o__Xanthomonadales; f__Sinobacteraceae; g__; s__ | 0.008% | 0.008% | 0.003% |
| k__Bacteria; p__Proteobacteria; c__Gammaproteobacteria; o__Xanthomonadales; f__Xanthomonadaceae | 0.222% | 1.690% | 0.434% |
| k__Bacteria; p__Proteobacteria; c__Gammaproteobacteria; o__Xanthomonadales; f__Xanthomonadaceae | 0.003% | 5.414% | 0.114% |
| k__Bacteria; p__Proteobacteria; c__Gammaproteobacteria; o__Xanthomonadales; f__Xanthomonadaceae | 0.000% | 2.953% | 0.079% |
| k__Bacteria; p__Proteobacteria; c__Gammaproteobacteria; o__Xanthomonadales; f__Xanthomonadaceae | 0.001% | 0.925% | 0.033% |
| k__Bacteria; p__Proteobacteria; c__Gammaproteobacteria; o__Xanthomonadales; f__Xanthomonadaceae | 0.000% | 0.010% | 0.004% |
| k__Bacteria; p__Proteobacteria; c__Gammaproteobacteria; o__Xanthomonadales; f__Xanthomonadaceae | 0.000% | 0.051% | 0.002% |
| k__Bacteria; p__Proteobacteria; c__Gammaproteobacteria; o__Xanthomonadales; f__Xanthomonadaceae | 0.000% | 0.054% | 0.002% |
| k__Bacteria; p__Proteobacteria; c__Gammaproteobacteria; o__Xanthomonadales; f__Xanthomonadaceae | 0.000% | 0.010% | 0.001% |
| k__Bacteria; p__Proteobacteria; c__Gammaproteobacteria; o__Xanthomonadales; f__Xanthomonadaceae | 0.000% | 0.079% | 0.001% |
| k__Bacteria; p__Proteobacteria; c__Gammaproteobacteria; o__Xanthomonadales; f__Xanthomonadaceae | 0.000% | 0.048% | 0.001% |
| k__Bacteria; p__Proteobacteria; c__Gammaproteobacteria; o__Xanthomonadales; f__Xanthomonadaceae | 0.000% | 0.011% | 0.001% |
| k__Bacteria; p__Proteobacteria; c__Gammaproteobacteria; o__Xanthomonadales; f__Xanthomonadaceae | 0.000% | 0.012% | 0.000% |
| k__Bacteria; p__Proteobacteria; c__Gammaproteobacteria; o__Xanthomonadales; f__Xanthomonadaceae | 0.000% | 0.016% | 0.000% |
| k__Bacteria; p__Proteobacteria; c__Gammaproteobacteria; o__Xanthomonadales; f__Xanthomonadaceae; g__; s__ | 0.000% | 0.536% | 0.271% |
| k__Bacteria; p__Proteobacteria; c__Gammaproteobacteria; o__Xanthomonadales; f__Xanthomonadaceae; g__Dokdonella; s__ | 0.013% | 0.033% | 0.012% |
| k__Bacteria; p__Proteobacteria; c__Gammaproteobacteria; o__Xanthomonadales; f__Xanthomonadaceae; g__Rhodanobacter | 0.000% | 1.031% | 0.139% |
| k__Bacteria; p__TM6; c__SJA-4; o__; f__; g__; s__ | 0.048% | 0.308% | 0.008% |
| k__Bacteria; p__Verrucomicrobia; c__Verrucomicrobiae; o__Verrucomicrobiales; f__Verrucomicrobiaceae; g__Akkermansia; s__muciniphila | 0.142% | 0.007% | 0.000% |
